# Investigating tumor immunogenicity in breast cancer: deciphering the tumor immune response to enhance therapeutic approaches

**DOI:** 10.3389/fimmu.2024.1399754

**Published:** 2024-10-23

**Authors:** Oumayma Naji, Amina Ghouzlani, Soumaya Rafii, Rizwan ullah Sadiqi, Abdou-samad Kone, Zakia Harmak, Khalil Choukri, Sarah Kandoussi, Mehdi Karkouri, Abdallah Badou

**Affiliations:** ^1^ Immuno-Genetics and Human Pathologies Laboratory (LIGEP), Faculty of Medicine and Pharmacy, Hassan II University, Casablanca, Morocco; ^2^ Faculty of Science and Technology, Middlesex University, London, United Kingdom; ^3^ Department of Pathological Anatomy, University Hospital Center (CHU) Ibn Rochd and Faculty of Medicine and Pharmacy of Casablanca, Hassan II University, Casablanca, Morocco; ^4^ Mohammed VI Center for Research and Innovation, Rabat and Mohammed VI University for Sciences and Health, Casablanca, Morocco

**Keywords:** breast cancer, immune microenviroment, immune response, therapy resistance, immunotherapy

## Abstract

The interplay between immune cells and malignant cells represents an essential chapter in the eradication of breast cancer. This widely distributed and diverse form of cancer represents a major threat to women worldwide. The incidence of breast cancer is related to several risk factors, notably genetic predisposition and family antecedents. Despite progress in treatment modalities varying from surgery and chemotherapy to radiotherapy and targeted therapies, persistently high rates of recurrence, metastasis, and treatment resistance underscore the urgent need for new therapeutic approaches. Immunotherapy has gained considerable ground in the treatment of breast cancer, as it takes advantage of the complex interactions within the tumor microenvironment. This dynamic interplay between immune and tumor cells has become a key point of focus in immunological research. This study investigates the role of various cancer markers, such as neoantigens and immune regulatory genes, in the diagnosis and treatment of breast tumors. Moreover, it explores the future potential of immune checkpoint inhibitors as therapeutically effective agents, as well as the challenges that prevent their efficacy, in particular tumor-induced immunosuppression and the difficulty of achieving tumor specificity.

## Introduction

Breast cancer ranks as the foremost-diagnosed cancer among women globally, posing a considerable threat to their health and well being. In 2020, there were reported 2.26 million new cases, with predictions indicating a 21% increase over the next decade. In the United States alone, it accounts for over 40,000 deaths annually (1).

Notably, it stands as not only the most common cancer in women but also the leading cause of cancer-related mortality worldwide. The disease resulted in 684,996 deaths globally [95% UI, 675,493–694,633], showcasing an age-adjusted rate (AAR) of 13.6 per 100,000 (2). Despite the higher incidence rates in developed regions, 2020 witnessed 63% of breast cancer deaths occurring in Asia and Africa. The survival rate among women diagnosed with breast cancer in high-income countries contrasts sharply with that of women in low- and middle-income countries, where the prognosis is often less favorable (3). Analysis of gene expression arrays has led to the recognition of several essentially different subtypes of breast cancer (4, 5). According to the St. Gallen Consensus 2011, molecular subtypes of breast cancer can be classified into Luminal Type A which is characterized by being progesterone receptor (PR) positive, estrogen receptor (ER) positive, human epidermal growth factor receptor 2 (HER2) negative, and lowKi-67; Luminal B is PR positive, ER positive, may be either HER2 positive or negative, and high Ki-67. HER2-overexpressing subtypes are ER negative, PR negative, and HER2 positive, while triple-negative breast cancer (TNBC) lacks expression of ER, PR, and HER2 (6).

Breast cancer subtypes differ according to their degree of immunogenicity, which is very important with respect to treatment strategies. It has been reported that luminal A breast cancer shows usually low immunogenicity, characterized by low tumor-infiltrating lymphocytes (TILs) T and low PD-L1 expression, hence suggesting a low immune response (7, 8). In contrast to luminal A, the luminal B breast cancer subtype seems more immunogenic and has greater T-cell clonality, but still represents a medium level of immune response with reduced levels of CD8+ TILs (9, 10). HER2-positive breast cancer also appears more immunogenic, often expressing high levels of TILs and PD-L1 expression that generally correlated with better immunotherapy responses and improved survival (11). Additionally, Triple-negative breast cancer has been noted as the most immunogenic subtype of breast cancer, distinguished by a high level of TILs and PD-L1 expression. TNBC patients are significantly more responsive to treatment with immunotherapy (8). Given its high mutational burden and high immune infiltrate, TNBC represents a promising target for immunotherapy. Indeed, in both early and advanced stages of TNBC cases, the high presence of tumor-infiltrating lymphocytes (TILs) is a predictor of favorable responses to immunotherapy, contributing to a better outcome in terms of survival (8). Compared to other forms of breast cancer, TNBC tends to grow and spread more rapidly. Patients with Luminal A and B, as well as HER2-enriched subtypes, show responsiveness to targeted therapies, whereas those with the triple-negative phenotype typically exhibit a poorer prognosis (12). This sophisticated categorization aids in tailoring treatment approaches and enhancing patient outcomes, underscoring the critical nature of ongoing research and intervention strategies in combating this pervasive disease (13). In clinical practice, immunohistochemical analyses of tumors are performed on the basis of ER, PR and HER2 status. This method is simpler and less expensive and provides similar results for molecular subtypes (14).

Therefore, incorporating molecular subtyping techniques into clinical practice is crucial as it offers precise information regarding a patient’s prognosis, relapse risk, and likelihood of achieving a complete pathological response. This approach facilitates the identification of patients who would benefit most from neoadjuvant therapy, thereby improving risk stratification. Moreover, by identifying specific molecular subtypes and breast cancer markers, clinicians can tailor treatment modalities that target the underlying biological characteristics of the tumor, leading to more effective therapies. This not only enhances treatment outcomes but also minimizes exposure to treatments that may not be beneficial for certain subtypes. Additionally, molecular subtyping allows for the development of more aggressive treatment strategies or enhanced surveillance for patients with a higher risk of relapse (15). Additionally, it allows for the development of more aggressive treatment strategies or enhanced surveillance for patients with a higher risk of relapse (16, 17). The latest edition of the Tumor–Node–Metastasis (TNM) classification introduces an updated staging methodology that considers not only the anatomical features of breast cancer but also its biological attributes (18).

Breast cancer treatment is comprehensive, embracing various modalities such as surgery, radiation therapy, chemotherapy, hormonal therapy, and biological therapies. These treatments are applied in different sequences, tailored to the individual patient’s condition (19).

This review article aims to shed light on the immune microenvironment within breast cancer, emphasizing novel therapeutic strategies that may enhance the efficacy of immunotherapy and address resistance issues.

## Immune response in breast cancer

In the initial phases of tumor development, components of the host’s immune system, especially cells of the innate immune system, play a pivotal role in eradicating tumor cells, including those associated with breast cancer (20).

The innate immune response, triggered by various innate immune cells without the need for prior sensitization, possesses the capacity to promptly neutralize cancer cells (21–23). Moreover, this response can precipitate the activation of enduring adaptive immune responses, thereby enhancing the efficacy of tumor cell destruction (24, 25). Given these dynamics, targeting the modulation of innate immunity emerges as a compelling strategy for cancer therapy. This chapter will focus on the role of innate immune cells, alongside identifying therapeutic targets and pathways, with the aim of advancing the development of translational anticancer therapies.

## Macrophages

Macrophages are a key cellular component of innate immunity, primarily recognized for their phagocytic capabilities (26). In addition to tumor cells, the tumor microenvironment comprises various immune cells, including macrophages. Macrophages exist in different activation states, with M1 and M2 representing two distinct phenotypes (26). In non-pathological state, macrophages present within tissues are tissue-resident macrophages and/or monocyte-derived macrophages. These macrophages maintain tissue homeostasis by performing numerous functions like immunosurveillance, clearance of senescent and apoptotic cells, and maintenance of tissue architecture (27).

Macrophages polarize and develop diverse properties in response to a variety of signals. Exposure of macrophages to Interferon-gamma IFN-γ and lipopolysaccharide (LPS) induces M1 polarization, with cytotoxic and antitumor properties potentiated, in contrast to M2 macrophages that are more likely to have immunoregulatory and protumor activities. In particularly, M2a macrophages (‘ induced by exposure to Interleukin-4 (IL-4) and Interleukin-13 (IL-13)) and M2b macrophages (‘ induced by combined immune complex exposure and toll-like receptors (TLR) or IL-1R agonists) exert immunoregulatory functions and induce type II responses, whereas M2c macrophages (‘ induced by Interleukin-10 (IL-10)) are more associated with suppression of immune responses and tissue remodeling (28).

**Figure 1:**
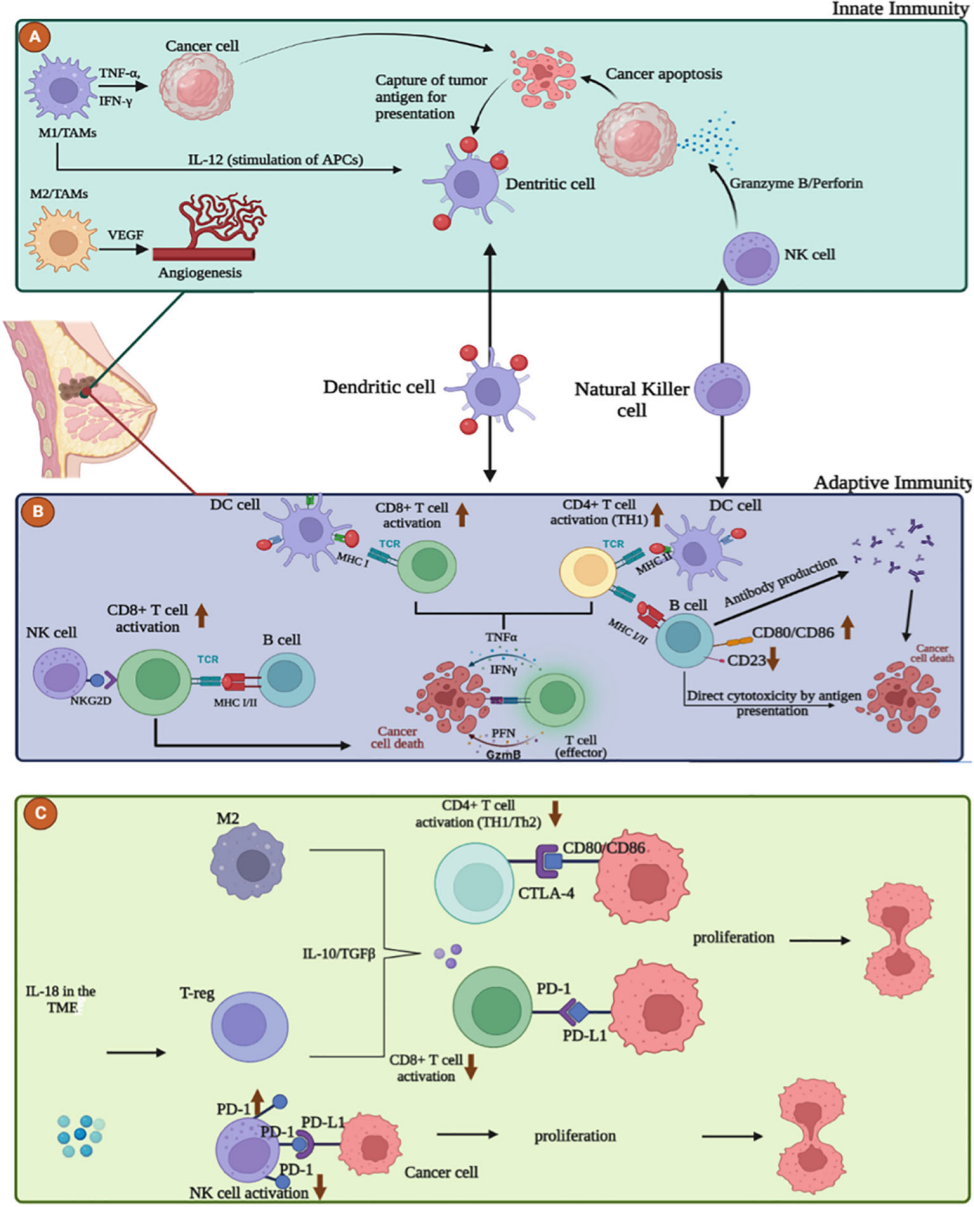
The interplay between the immune system and breast cancer microenvironment (TME). **(A)** Role of innate immune cells in breast cancer microenvironment. M1 macrophages and Natural killer cells act as anti-tumor cells by releasing stimulatory molecules such as TNFα, IFNγ, IL-12 and cytotoxic granules such as Perforin, Granzyme B that destroy cancer cells. M2 macrophages promote resistance, immunosuppression and tumor progression by angiogenesis via VEGF production. **(B)** Adaptive anti-tumor response in the breast cancer microenvironment. Maturation and activation of CD8+ and CD4+ T cells can occur through uptake and presentation of tumor antigens by dendritic cells, B cells, via MHC I/II, but also through the interaction between the NKG2D receptor on NK cells and the NKG2D ligand on T cells. Activated effector T cells destroy cancer cells by secreting Perforin, Granzyme, TNFα and IFNγ. B cells can exert direct cytotoxicity after recognition of tumor antigen or antibody-dependent cell-mediated cytotoxicity. **(C)** Adaptive pro-tumor response in the breast cancer microenvironment. Inhibition and exhaustion of CD8+ and CD4+ T cells can occur through the secretion of anti-inflammatory cytokines such as TGF-β and IL-10 by M2 macrophages and T-reg and also through the high expression of PD-1, CTLA-4 on NK and T cells.

In the context of breast cancer, macrophages are found in greater numbers within cancerous tissues compared to adjacent non-cancerous tissues. They are associated with epithelial-to-mesenchymal transition markers, which are implicated in the progression and metastasis of breast cancer (26, 29). The colony stimulating factor 1 (CSF-1), a critical regulator of macrophages, is linked with a poor prognosis in breast cancer patients (30). Furthermore, an increase in M2 macrophages correlates with a decrease in survival rates among these patients (31, 32). Macrophages also play a role in neovascularization. Studies have shown that the secretion of vascular endothelial growth factor (VEGF) by macrophages is two to three times higher than that of breast cancer cells themselves (33). M2 macrophages, in particular, contribute to pro-tumorigenic Th2/humoral responses and immunosuppression (**Figure 1A**). Conversely, stimulation of human macrophages towards an M1 phenotype exhibits an antitumor effect (34). A high presence of M2-type macrophages has been observed in DNA-damage immune-response (DDIR)-negative tumors, which are characterized by an immunosuppressive TME, unlike DDIR-positive tumors (35). Additionally, the macrophage receptor tyrosine kinase, c-Mer (Mertk), is associated with poor prognosis due to its role in fostering an immunosuppressive environment (36, 37). However, interventions using Mertk-knockout mice or neutralizing anti-Mertk antibodies have been shown to alter the immune response profile, leading to an increase in inflammatory factors, greater T-cell infiltration into tumors, and enhanced cytotoxicity (37). Recently, there have been many studies about the involvement of macrophages in tumors. Among the main concerns in this area is the origin and function of Tumor Associated macrophages (TAMs). TAMs can increase the growth, invasion and metastasis of tumor cells, as well as stimulate angiogenesis and suppress the T cell-mediated immune response against tumors, thereby promoting tumor progression (38, 39). Much clinical data show that more accumulation of TAMs in the tumor tissue indicates a poor prognosis for cancer patients (40).

However, there are conflicting reports on the cellular origin of TAMs that accumulate in the tumor microenvironment. In a subsequent issue of Science, Franklin et al. showed that TAMs in mouse models of breast cancer are phenotypically and functionally distinct from traditional M2 TAMs. Otherwise, TAMs differentiate from CCR2+ inflammatory monocytes and their differentiation relies on the Notch signaling pathway (41).

TAMs contribute to tumor progression by promoting angiogenesis, inhibiting T cells, and increasing the secretion of immunosuppressive cytokines (36) (**Figure 1C**). Overexpression of B7-H3 in TAMs plays a significant pro-metastatic and immunosuppressive role by remodeling the extracellular matrix and enhancing tumor angiogenesis, thereby facilitating tumor cell dissemination and reducing T-cell infiltration into the TME (42). Additionally, TAMs activate the nuclear factor kappa B (NF-κB)/SOX4 signaling pathway through the release of CXC motif chemokine ligand 1 (CXCL1), promoting epithelial-to-mesenchymal transition (EMT) and lung metastasis in breast cancer (43, 44).

## NK cells

The innate immune system plays a pivotal role in cancer defense, with effector lymphocytes such as natural killer (NK) cells providing critical, albeit transient, protection against malignancies. NK cells are adept at identifying and neutralizing target cells that express major histocompatibility complex I (MHC I) or MHC I-like molecules (45).

In breast cancer patients, the concentration of NK cells within tumor tissue is significantly reduced compared to their prevalence in the bloodstream (46), and a decrease in patient survival correlates with an increase in activated NK cells (31, 32). Activation of NK cells has proven effective in eradicating breast tumors (47), whereas suppression of these cells facilitates immune evasion and metastasis (48). Interleukin-22 (IL-22), for instance, upregulates CD155 expression. This interaction with the activating receptor CD226 on NK cells results in inhibition, thereby exacerbating the metastatic burden (49). To combat cancer cells, NK cells exhibit a CD3-CD56+CD16+ phenotype and secrete granules filled with perforins and granzymes (50, 51). Granzyme B, in particular, serves as a vital effector for NK cells, facilitating the elimination of breast cancer cells by reactivating p53 (52, 53). Moreover, NK cells can promote T cell maturation and activation through various cell surface receptors and cytokines (54). Interferon (IFN) also exhibits antitumor activity by stimulating both innate and adaptive immune responses, including enhancing NK cell activity (55, 56). Targeting Induction of Lectin-like Transcript 1 (LLT1), a ligand on breast tumors that interacts with NK cell receptors, through antibody blocking or gene knockdown disrupts this interaction, thereby augmenting the destruction of breast cancer cells by NK cells (36, 57). Additionally, Interleukin-18 (IL-18) within the TME can elevate programmed cell death 1 (PD-1) expression on NK cells, leading to a significant immunosuppressive effect (58). The microRNA miR-519a-3p shields breast cancer cells from NK cell-mediated destruction and augments resistance to apoptotic death, further contributing to immune evasion (59). Certain chemotherapy agents directly influence NK cells. *In vitro* studies have shown that pretreatment with epirubicin significantly enhances NK cell-mediated cytotoxicity against tumor cells. This suggests that combining anthracycline-based chemotherapy with NK cell-based immunotherapy could be a potent strategy for breast cancer treatment (60). Initially, cytotoxic chemotherapeutics were found to impair NK cell responses in breast cancer patients (61, 62), though NK cell counts (CD56) typically normalize post-adjuvant chemotherapy (63). Similar observations have been reported in mouse models of solid tumors, including breast cancer, where there is a reduction in peripheral and tumor-infiltrating mature NK cells essential for antibody-dependent cell-mediated cytotoxicity (ADCC) and an increase in the expression of inhibitory receptors on tumor cells that block ADCC (64). Other mechanisms of suppression involve the expression of CD39 on breast cancer cells, which interacts with regulatory NK cells expressing CD73 infiltrating the tumor. Through the activation of signal transducer and activator of transcription-3, CD73+ NK cells induce IL-10 production, which hampers the proliferation of CD4+ T cells and IFN-γ production, leading to immune tolerance (65). Breast cancer cells expressing the epithelial cell adhesion molecule are more effectively eliminated by IL-15 CAR NK cells compared to NK-resistant types (66). The enhanced ADCC activity of human NK cells is attributed to increased binding affinity to Fc gamma receptor IIIa (FcRIIIA), inhibiting cell proliferation (67). Margetuximab’s modified binding characteristics significantly enhance ADCC and anti-tumor effects, especially against cells with low HER2 levels or those resistant to trastuzumab (68). Furthermore, telomerase inhibition (hTERT) has been demonstrated to augment the sensitivity of breast cancer cells to NK cell therapy, enhancing NK cell cytotoxicity (69). Additional research indicates that NK cells, when combined with the RuPOP (ruthenium (Ru) polypyridyl) molecule, can produce significant amounts of reactive oxygen species (ROS), activate apoptosis-related receptors such as TNF-R1, DR5, and Fas, and improve interactions between NK and tumor cells by upregulating The Natural Killer Group 2D (NKG2D) and its numerous ligands, leading to caspase 3-dependent apoptosis (70). This combination therapy not only increases NK cell infiltration but also reduces the pro-tumoral capacity of myeloid-derived suppressor cells (MDSC), thereby achieving high therapeutic efficacy against breast tumors *in vivo* (70).

## Dendritic cells

Dendritic cells (DCs) serve as pivotal antigen-presenting cells that bridge innate and adaptive immunity. They play a crucial role in the TME immunosurveillance, exhibiting a marked propensity to infiltrate TNBCs more than other subtypes, which correlates with favorable clinical outcomes in breast cancer (71–75). However, an increased presence of plasmacytoid DCs (pDCs) within breast cancer has been linked to poor clinical prognosis (76, 77). The maturation of DCs significantly enhances cytotoxic T lymphocyte (CTL) responses, positively influencing tumor suppression efforts (78). Immunotherapy leveraging mature DCs has been shown to augment the populations of CD8+ and CD4+ T cells, curb the growth of breast tumors through the induction of apoptosis and anti-mitotic mechanisms, and thwart metastasis by reducing the expression of the transcription factors Snail, Slug, and Twist (79). Furthermore, the maturation of dendritic cells induced by a compound known as shikonin notably improved CTL responses, offering substantial benefits in tumor control. This suggests that DC-based vaccines could represent a promising treatment modality for breast cancer (BC) patients who have become resistant to chemotherapy. The use of tumor lysate from individuals with locally advanced BC has proven effective in consistently activating CD8+ CTLs, targeting and eliminating cancer cells (80). Additionally, the combination of doxorubicin and cyclophosphamide with autologous DCs has been successful in prolonging T cell longevity and revitalizing immune functionality (81, 82).

## Adaptive immune responses

The adaptive immune response plays a pivotal role in the immune system, orchestrating targeted and specific recognition and elimination of pathogens and foreign substances, as well as modulating responses to treatment. Recent studies have illuminated the adaptive immune response’s potential influence on the development and progression of cancer, with a particular focus on breast cancer. This research suggests that the adaptive immune response can have dual outcomes—either beneficial or detrimental—contingent upon various factors, such as the cancer’s type and stage (83). Distinct from the innate immune system, the adaptive immune system is characterized by its capacity to remember previous encounters, providing both humoral and cellular immunity via B and T cells, respectively. In the context of oncology, the adaptive immune system is heralded for its potential to elicit long-term and efficacious responses (84).

Moreover, the adaptive immune response to breast cancer involves a complex interplay among various key entities, each assuming critical and diverse roles. This ensemble includes T cells, B cells, natural killer cells, and dendritic cells, collaborating to identify and eradicate cancerous cells. Research indicates a positive correlation between the infiltration of T cells within breast tumors and improved prognoses and treatment outcomes (85). Additionally, therapeutic interventions that target the adaptive immune response, such as immune checkpoint inhibitors, have shown promise in treating breast cancer{Citation}Comprehending the multifaceted nature of this response is imperative for the development of innovative treatments capable of leveraging the immune system’s robustness against breast cancer.

## CD8 T cells

Based on their distinct phenotypes and roles, CD8 T cells constitute a key subset of T lymphocytes in adaptive immunity, serving as the primary effector mechanism responsible for initiating anti-tumor immune responses (**Figure 1B**). These cells are further delineated into two subsets (86): Cytotoxic T cells (Tc), which act as the principal agents of cell-mediated destruction within adaptive immunity (87). Upon engagement with major histocompatibility complex class-1 (MHC-1) molecules on antigen-presenting cells (APCs), these cells release effector cytokines, including interferon-γ (IFNγ) and TNF, along with cytotoxic molecules such as granzymes and perforin. Consequently, they are adept at targeting and eliminating malignancies, positioning them as formidable adversaries against tumor cells (88). The other subset, CD8 regulatory T cells (Treg) plays an essential role in moderating immune responses by dampening Th cell activity and curtailing immune reactions to infections (87). Recent studies have correlated the presence or absence of tumor-infiltrating CD8+ T cells within the TME with various stages of tumor development and prognoses across numerous cancers (89–92). Specifically in breast cancer, these T-cell subsets demonstrate highly synchronized roles and distinct functionalities within the adaptive immune system. Elevated levels of CD8+ T-cell infiltration have been associated with favorable prognostic indicators, including enhanced overall survival (OS) and disease-free survival (DFS) in patients with breast cancer. Moreover, a significant association was found between increased CD8+ T-cell infiltration and lower expression levels of the estrogen and progesterone receptors, alongside heightened expression of HER2, a recognized oncogenic protein. These findings underscore the pivotal role of CD8+ T-cells in mounting an immune response against breast cancer and their potential utility as prognostic markers for patient outcomes (93). However, the presence of CD8+ T cells alone does not guarantee tumor regression, as tumors expressing highly immunogenic neoantigens can still progress (94). Breast cancer, like other malignancies, can foster an immunosuppressive TME and develop resistance to the antitumor activity of CD8+ T cells, thereby diminishing their therapeutic efficacy (95). The TME promotes the accumulation of TAMs, Tregs, and MDSCs by secreting immunosuppressive cytokines, which in turn impairs CD8+ T cell infiltration, proliferation, and function within the tumor (83). Furthermore, tumor-infiltrating CD8+ T cells in human breast cancer exhibit increased programmed death-ligand 1 (PD-L1) expression (**Figure 1C**), correlating with compromised immune functionality and subsequent T cell suppression (96). The upregulation of PD-L1 on breast cancer cells is linked to the inhibition of dendritic cell maturation and reduced T cell tumor infiltration, facilitated through the interaction of tumor PD-L1 with PD-1 or B7-1 receptors on T and B cells (97, 98). Previous research has demonstrated the significance of assessing PD-L1 expression and CD8+ lymphocyte infiltration in TNBC, offering valuable prognostic and predictive insights (99).

The compelling correlation between the proportion of CD8+ lymphocytes and PD-L1 expression suggests that the analysis of CD8+ T-cell infiltrates could act as a complementary or alternative biomarker for PD-L1. This potential allows for the improved identification of patients who are most likely to benefit from immunotherapy (100).

Nonetheless, the observed lack of tumor specificity by CD8+ tumor-infiltrating lymphocytes (TILs) in breast tumors may account for the modest clinical responses observed with checkpoint blockade therapies (101). A recent study has demonstrated that bi-specific antibodies can enable CD8+ TILs from human breast tumors to effectively eradicate cancer cells. Reports have indicated that CD8+ TILs in human breast tumors retained their polyfunctionality, even while expressing PD-1, and propose their significant utility in potent immunotherapies (102).

## CD4 T cells

CD4+ T cells exhibit remarkable versatility and polyfunctionality, serving as a vital component of adaptive T cell immunity, working in concert with their CD8+ cytotoxic T cell counterparts (103). These cells are adept at recognizing antigens in the context of MHC class II molecules, which are primarily expressed on immune cells. Notably, conventional T (T_conv) cells predominantly exert their immunomodulatory effects by recognizing antigens presented on specialized antigen-presenting cells, such as dendritic cells (DCs) and macrophages (104).

Upon receiving context-dependent signals, CD4+ T cells can differentiate into diverse functional subtypes. This differentiation enables them to play a central role in coordinating the immune response, assisting appropriate effector immune cells in their functions (105). The primary function of CD4+ T cells includes facilitating anti-tumor immunity through various mechanisms. These mechanisms include supporting CD8+ cytotoxic T cells and antibody responses, secreting cytokines such as interferon-γ (IFNγ) and tumor necrosis factor-α (TNFα), and, under specific conditions, directly targeting tumor cells with cytotoxic activity (106).

**Figure 2:**
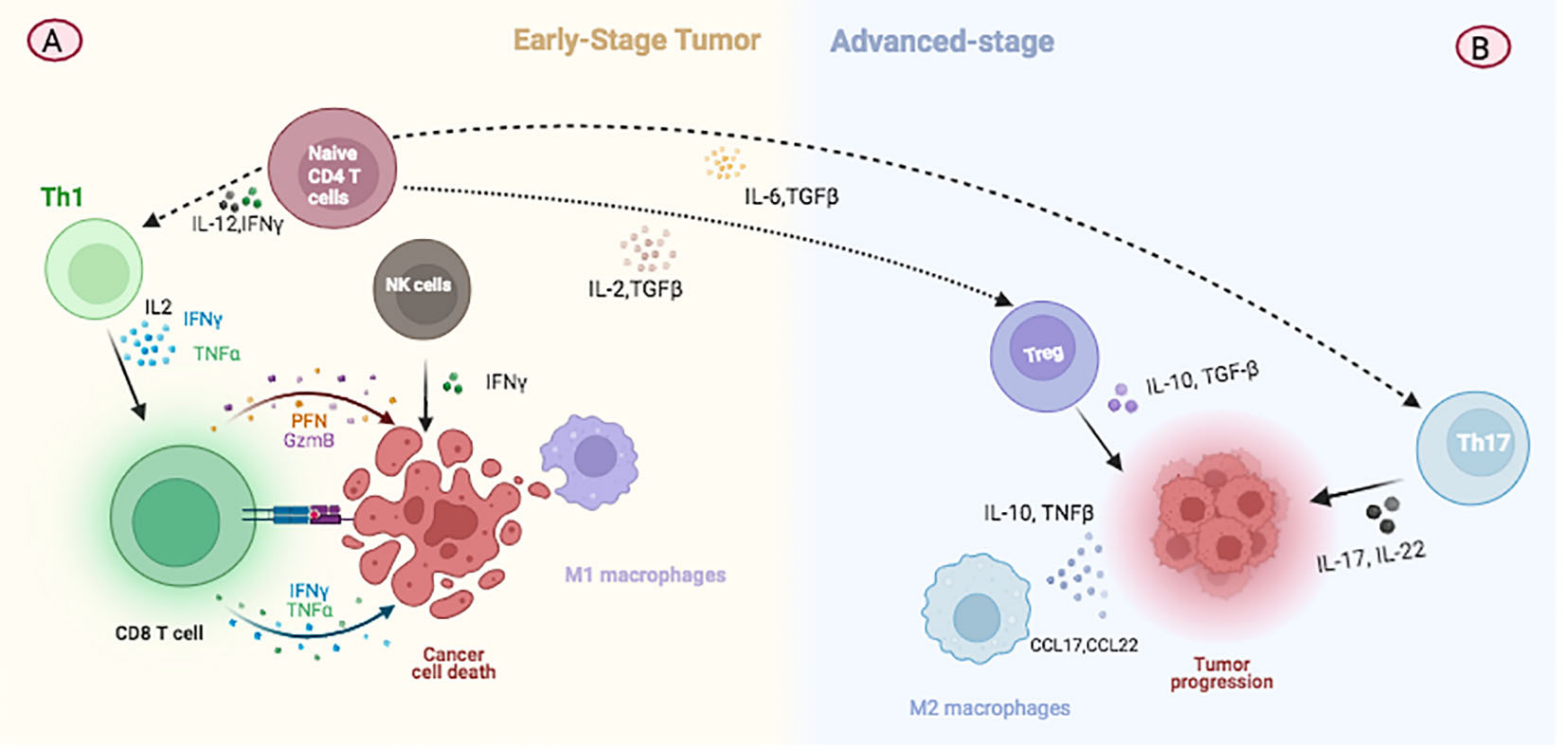
Illustration depicting changes in immune cell populations during breast cancer progression. **(A)** At the initial stage of tumor development, TILs predominantly consist of Th1 and CD8+ T cells which are involved in immunosurveillance and combating malignant cell growth. **(B)** In advanced stages of cancer, there is a notable increase in CD4+ TILs, with a shift towards the predominance of Treg and Th17 cells. These changes contribute to tumor growth by modulating the immune environment within the tumor.

In the tumor environment and in the circulation of patients, CD4+ cytotoxic T lymphocytes (CTLs) express cytolytic effector molecules, including granzymes, perforin, and other granule-associated proteins such as natural killer cell granule Protein 7 (NKG7) and granulysin (107). It has been consistently documented that CD4+ T cell help is essential for inducing and maintaining functional memory CD8+ T cell responses (108). The role of CD4+ T cells in tumor defense has been extensively studied in animal models prior to clinical trials, as well as in patients with cancer (109). Further investigations in patients have revealed the expansion of CD4+ CTLs in various tumor types, including breast cancer (110). Huang and colleagues have highlighted the dynamic changes within tumor-infiltrating lymphocyte (TIL) populations during the progression of breast cancer. Their research indicates that, during the initial stages of tumor development, TILs predominantly consist of Th1 and CD8+ T cells, potentially performing immunosurveillance to combat malignant cell growth (**Figure 2A**). Conversely, in advanced stages of cancer, there is a notable increase in CD4+ TILs, with a shift in dominance to Treg and Th17 cells, which may facilitate tumor growth (**Figure 2B**) (109). Significantly, the effectiveness of targeted therapies is markedly improved by the presence of CD4+ T cells, suggesting that trastuzumab may elicit immune-mediated cytotoxicity. The increased expression of TNF-α receptors implies that cytokines may synergize with trastuzumab, enhancing responsiveness to therapy in HER2+ breast cancer and potentially reducing tumor burden (111).

Importantly, Th2 cell immunity, facilitated by the release of interleukin 3 (IL-3), interleukin 5 (IL-5), and granulocyte-macrophage colony-stimulating factor (GM-CSF), induces terminal differentiation in developing breast cancer. This process leads to the reversal of high-grade breast cancers into low-grade, fibrocystic-like structures. These findings underscore the critical role of CD4+ Th2 cells in immune response against breast cancer, highlighting terminal differentiation as a distinct mechanism for cancer immunoprevention and therapy (112).

Moreover, Lhuillier et al. have demonstrated that radiation therapy (RT) increases the expression of genes responsible for the generation of immunogenic neoepitopes and stimulates CD4+ T cell responses in a mouse model of triple-negative breast cancer. Importantly, neoantigen-specific CD4+ T cells are capable of producing Th1 cytokines, eradicating irradiated tumor cells, and facilitating epitope spreading. These findings highlight the pivotal role of RT as a complementary approach to neoantigen vaccination, enhancing vaccine efficacy (113).

The activation of CD4+ T cells for cancer immunoprevention and therapy offers several distinct advantages over conventional immunotherapies that target CD8+ T cells. As upstream activators of adaptive immunity, CD4+ T cells can directly activate and target tumor antigens, initiating a robust anti-tumor immune response in “cold” tumors, which include early-stage epithelial cancers and precancerous lesions (105). In summary, cytotoxic CD4+ T cells emerge as a promising prognostic biomarker within the immune microenvironment of breast cancer. A detailed exploration of the activities of CD4+ cytotoxic T lymphocytes could provide a predictive marker for immunotherapy in breast cancer patients.

## B-cells

After activation in the germinal centers of lymphoid organs, B cells that express high-affinity antibodies differentiate into plasma cells, which secrete antibodies, and memory B cells, which are crucial for driving humoral immunity against pathogens (114). Notably, B cell activation occurs specifically within the germinal centers of lymphoid organs (115).

In addition to their ability to produce cytokines and differentiate into plasmablasts, B cells also function as antigen-presenting cells (APCs) by presenting antigens to T cells (**Figure 1B**). As APCs, B cells typically show increased expression of costimulatory proteins essential for T cell activation (116).

Within the TME, B cells exhibit distinct surface marker expression profiles compared to those circulating in peripheral blood. There is a marked upregulation of costimulatory proteins, such as CD86, and a reduction in CD23 expression, indicating higher activation levels of B cells within the TME (117).

In breast cancer patients, tumor-infiltrating B cells (TIL-B) have been identified in up to 60% of cases (116). Furthermore, B cell infiltration has been consistently associated with a favorable prognosis in breast cancer, as evidenced by various studies (118, 119).

Significantly, memory B cells exhibit a markedly higher prevalence in breast cancer tissues compared to healthy tissues. Their presence consistently correlates with positive outcomes and enhanced responses to chemotherapy, especially in highly proliferative breast cancer subtypes, such as triple-negative breast cancer (116, 120). Notably, through the application of immunohistochemistry for B cell detection or immunogenomics for identifying B cell metagene signatures, researchers have established a link between improved prognosis and increased B cell infiltration in breast cancer patients (121). Recent preclinical studies on triple-negative breast cancer models have highlighted the critical role of B cell-mediated T cell activation and antibody production in the response to immune checkpoint inhibitors (ICI) in murine models with high-mutational-burden triple-negative breast cancer. Moreover, the effectiveness of dual checkpoint blockade significantly decreases following B cell depletion or in murine models lacking the ability to produce antibodies (122). Emerging evidence points to a unique subset of B cells, known as regulatory B cells (Bregs), which play a pivotal role in modulating the anti-tumor immune response. Bregs, characterized as CD1d+ CD5+ CD19+ immunoregulatory B cells producing IL-10, are analogous to Tregs in maintaining a balance between self-tolerance and immune activation (120).

In murine models, the 4T1 breast cancer line triggers the proliferation of tumor-associated Bregs (123), which suppress immune responses by secreting anti-inflammatory mediators such as interleukin 10 (IL-10), interleukin 35 (IL-35), and transforming growth factor beta (TGF-β). These mediators facilitate the conversion of T cells into Tregs, further illustrating the intricate interplay between Bregs and the immune system’s regulation (124).

In 2019, a study examining breast cancer patients revealed that the presence of IL-10+ Bregs was augmented in parallel with Tregs within primary tumors, correlating with shorter relapse-free intervals (125). Another study demonstrated that the accumulation of Bregs was linked to the infiltration and regulation by CD33+ MDSCs, contributing to an immunosuppressive TME (126). Furthermore, researchers have discovered an increase in Bregs expressing CD19+, CD24+, and CD38+ and producing IL-10 in breast cancer patients. Additionally, this population of Bregs was found to overexpress PD-L1, a ligand for the PD-1 receptor (127).

The coexistence of PD-L1+CD19+CD24+CD38+ Bregs exhibited a significant association with CD4+FoxP3+CD127low/- Tregs and a lower clinical survival rate. This suggests a possible feedback mechanism in which IL-10 production by Bregs promotes the generation of innate immune cells that enhance the persistence or quantity of Tregs in the TME (128).

Overall, a series of recent clinical studies suggests that the infiltration of B cells and plasma cells, as well as the isotype antibodies produced by these cells in the TME, are consistently associated with better outcomes and superior responses to existing immunotherapies. In fact, the predictive value of T cell infiltration is only significant when it is concurrently associated with an accumulation of B cells in certain tumors (129).

## T regulatory cells

The presence of Treg cells within tumors is associated with both beneficial and detrimental outcomes for cancer patients. The increase in Treg cells within a tumor may suggest a sustained anti-tumor T-cell response, which could ultimately be suppressed. Treg cells utilize various mechanisms of immunosuppression to maintain control over these responses (130).

In breast cancer patients, Tregs with immunosuppressive capabilities are significantly concentrated, and higher levels of intratumoral Tregs correlate with increased tumor grade and decreased survival rates (131, 132).

Furthermore, clinical evidence suggests that primary breast tumors influence Tregs beyond the TME, with an observed increase in Tregs in the peripheral blood of breast cancer patients (133). The responsiveness of these cells to cytokines is indicative of the likelihood of breast cancer recurrence (134). Additionally, recent studies have demonstrated the accumulation of Tregs in the sentinel lymph nodes of breast cancer patients, a factor associated with the spread of cancer to these nodes, suggesting a potential role for Tregs in the metastatic process (134, 135).

The TME redirects T cells capable of destroying tumors towards a regulatory function and promotes interleukin 17 (IL-17) production by Tregs through the secretion of various molecules, including microRNAs, cytokines, and extracellular vesicles, comprising the TME secretome. This secretome significantly influences the recruitment, differentiation, and polarization of Tregs through complex interactions (136).

Breast cancer patients exhibit at least two types of Tregs, differentiated by their site of maturation: naturally occurring CD4+CD25+ Tregs (nTregs) in the thymus and inducible Tregs (iTregs) in peripheral tissues. Additionally, IL-17-producing Tregs represent a transitional phase between Tregs and Th17 cells. While nTregs modulate other effector and immune cells through direct contact, iTregs do so by releasing anti-inflammatory cytokines such as TGF-β and IL-10. IL-17-producing Tregs suppress the immune system by secreting IL-10 and pro-inflammatory cytokines like interleukin 6 (IL-6) and IL-17. A small subset of CD4+CD25+FOXP3+ nTregs developed in the thymus plays a critical role in preventing autoimmunity. iTregs are pivotal in protecting against chronic inflammatory conditions and are believed to regulate immune responses to commensal microorganisms, maintaining peripheral tolerance and preventing local inflammation in response to external antigens (136).

In a mouse model replicating human metastatic breast cancer, recent research has documented the systemic activation and accumulation of highly immunosuppressive Tregs during the growth of primary tumors. These Tregs undergo transcriptional modifications in response to mammary carcinogenesis, exhibiting tissue-specific alterations. Furthermore, Tregs have been shown to modulate the activation of NK cells within lymph nodes, thereby promoting lymph node metastasis. This research demonstrates a heightened Treg/NK cell ratio in the sentinel lymph nodes of breast cancer patients as opposed to healthy individuals (135).

The elevation of Treg levels correlates with the absence of hormone receptor expression, lymph node metastases, and the presence of p53 and Ki-67 immunopositivity. In TNBC, a notable association exists between the extensive infiltration of immunosuppressive Tregs and the mutation of BRCA1. Moreover, the presence of Tregs within the TME is related to the histologic subtype and tumor grade, both recognized prognostic indicators. Consequently, targeting Tregs may offer a therapeutic advantage in improving the prognosis of TNBC treatment (137).

Significantly, following neoadjuvant chemotherapy, a complete pathological response is associated with a reduced abundance of Tregs, while a high abundance of Tregs correlates with elevated expression of checkpoint inhibitor genes in TNBC (138).

## Immunotherapy in breast cancer

Current breast cancer treatments include chemotherapy, hormonal therapy, radiotherapy, and tumor removal. Despite these methods, the pressing need for innovative treatment approaches is underscored by recent data. Recent advances in targeted therapies and immunotherapies offer promise, demonstrating effectiveness in treatment (139). Immunotherapy aims to stimulate the patient’s immune system or target specific components, including cytokines and monoclonal antibodies. The two most prevalent immunotherapies are checkpoint inhibitors and vaccines (140).

For decades, breast cancer was perceived as non-immunogenic. However, recent intensive research has firmly established the immune system’s dual role in breast cancer: it plays a crucial part in both suppressing tumor growth and promoting tumor progression (141). The complex and evolving relationship between the immune system and breast cancer has posed both challenges and successes. Recent focus has shifted towards immunotherapy in breast cancer, spurred by its success in improving survival rates in advanced cancers such as lung cancer, renal cancer, and melanoma. Consequently, immunotherapy for breast cancer has specifically targeted the immune checkpoint proteins PD-1 and cytotoxic T-lymphocyte associated antigen 4 (CTLA-4) (142).

**Figure 3:**
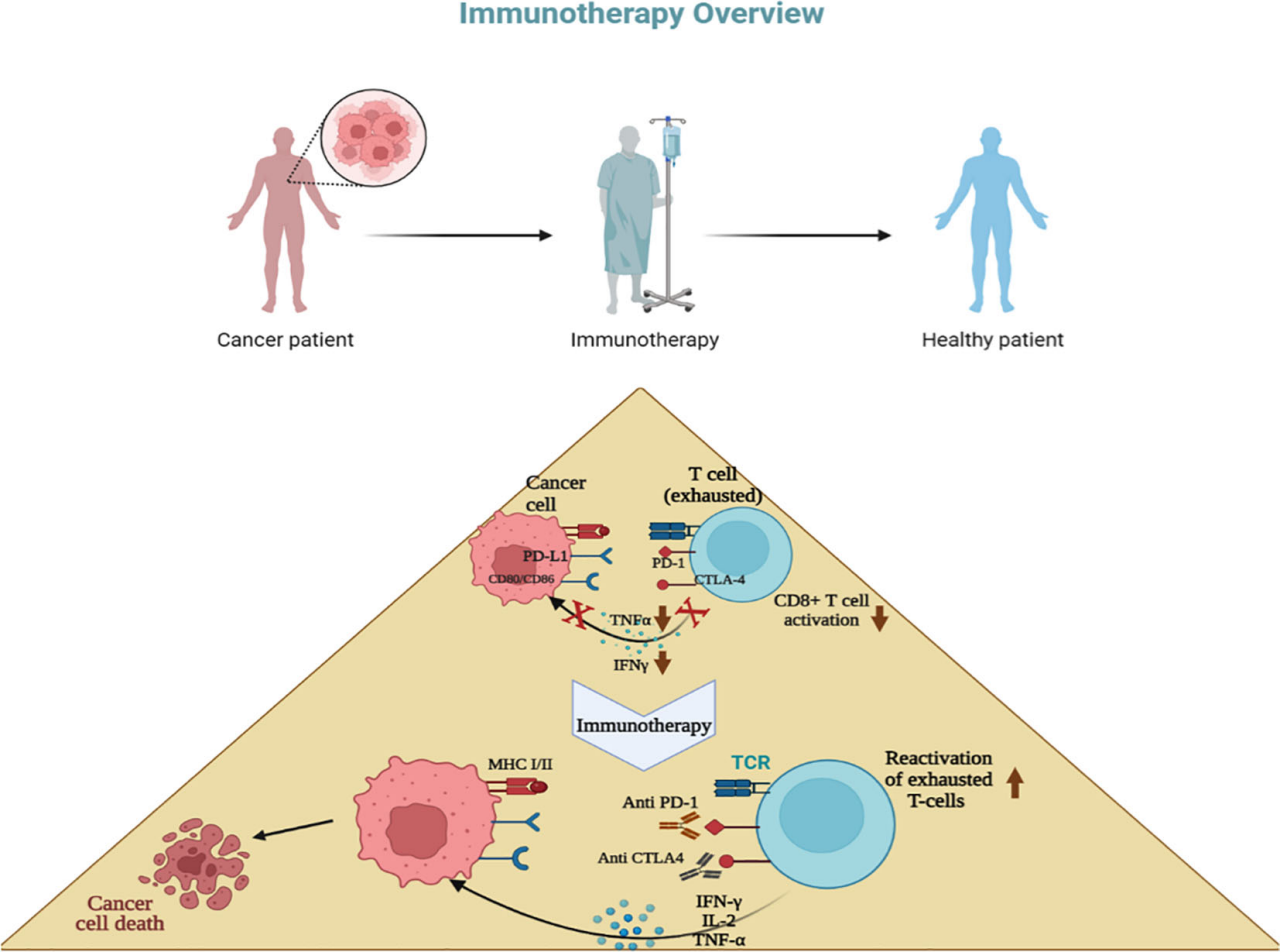
Concept of immunotherapy. T cells become exhausted after prolonged antigen stimulation and interaction with inhibitory ligands (PD-L1,L2; CD80, CD86) related to immune-checkpoint pathways. Immunotherapy involves inhibiting these immune checkpoint pathways using antibodies, with the goal of restoring T-cell functions. Most breast cancer immunotherapies focus mainly on anti-PD-1 drugs, which stop the interaction between PD-1, PD-L1, and PD-L2, such as pembrolizumab, avelumab, and atezolizumab.

The immune system’s core principle is to protect the body against infections and distinguish self from non-self. To prevent autoimmunity, checkpoints exist during the development of immune cells to identify and induce apoptosis in cells that may react against self-antigens. CTLA-4 and PD-1 are regulatory pathways expressed on many immune cells (**Figure 3**), which regulate the immune system by preventing excessive stimulation to self-antigens through inhibiting T-cell response. These pathways function at different stages of an immune response: CTLA-4 acts during the initial stages of T-cell development to eliminate potentially autoreactive T-cells, while PD-1, expressed on T-cells, B-cells, natural killer cells, and dendritic cells, functions during the effector phase at a later stage, primarily in peripheral tissues. Binding of PD-1 to its ligand PD-L1 activates downstream inhibition pathways, ultimately inhibiting T-cell activation. Therefore, PD-1 plays a critical role in preventing excessive immune stimulation by negatively regulating the immune response. However, it has been observed that abnormally high levels of PD-L1 expression on certain tumor cells, including those in breast and lung cancers, can lead to the downregulation of immune cells, aiding tumor cells in evading the immune system (143, 144).

Recent years have seen promising developments in cancer immunotherapy. The core principle of this approach is the inhibition of immune checkpoints, leading to PD-L1 dimerization and the dissociation of the PD-1/PD-L1 complex. This reactivates exhausted T-cells, enabling them to more effectively eliminate cancer cells. Consequently, the inhibition of these immune checkpoint pathways has resulted in the approval of several new drugs (141–146) (**Table 1**) and has proven to be successful in treating several types of cancers (90).

**Table 1:** Summary of Immunotherapy Approaches and trials results enrolling patient with Breast Cancer.

Drug Name	Mechanism of action	Target Immune Molecule	Breast cancer type/combined drug	Clinical trial results
Pembrolizumab	-Humanized monoclonal IgG4 antibody binds to the PD-1 receptor and prevents its communication with PD-L1 (147) -Increase T cell cytotoxicity against tumors by blocking PD-1 (147)	**PD-1**	Early TNBC+ + carboplatin + docetaxe	Phase III randomized KEYNOTE-522 clinical trial: -Pathological complete response -Approval of Neoadjuvant and adjuvant pembrolizumab (148)
			Advanced TNBC	Phase Ib KEYNOTE-012(NCT01848834) Antitumor efficacy in monotherapy (101)
			PD-L1-positive Metastatic TNBC	In the phase II KEYNOTE-086 trial: (NCT02447003), - ORR: 5.7% - Antitumor efficacy with a manageable safety profile (149)
Cemiplimab			High risk or progressive HR+ and HER_2_- breast cancer negative PD-L1+ or TNBC	A phase II clinical trial (NCT04243616) is ongoing to check therapeutic responses upon neoadjuvant chemotherapy and cemiplimab treatment (150)
			Early-stage BC + paclitaxel +anthracycline +cyclophospha-mide	NCT01042379 (ISPY): Pathological complete response (pCR) (150)
Nivolumab			Metastatic TNBC Anti-PD-1/L1 immunotherapy, alone or in combination with chemotherapy	Phase II study TONIC trial (NCT02499367): Upregulation of immune-related genes ORR: 20% 2 CRs and 11PRs (151)
Avelumab	- Human anti-PD-L1 IgG1 monoclonal antibody inhibiting the interaction between PD-1 and PD-L1 but not PD-1/PD-L2 (152) -Displays some antibody-dependent cell-mediated cytotoxicity (ADCC) (151)	**PDL-1**	Metastatic TNBC	JAVELIN (NCT01772004): -Antitumor activity was modest. -Objective response rate (ORR) was 5.2% (153)
			Metastatic breast cancer (MBC) with PD-L1+ or PD-L1− tumor-associated	JAVELIN phase Ib: -A trend toward higher ORR and stable disease. -Acceptable safety profile and clinical activity (153)
			High-risk TNBC after neoadjuvant (non pCR) or adjuvant (stage IIB–III) CT	A-Brave (NCT02926196): Ongoing study which compares 1 year of treatment with the anti PD-L1 avelumab vs observation for patients who completed treatment with radical intent for primary TNBC including surgery and chemotherapy (154)
Atezolizumab			Metastatic TNBC	Phase 1 study at US and European academic medical centers: - Manageable safety and tolerability -A high-level of infiltrating immune cells (>10%) which was associated with a better survival (155)
			Metastatic TNBC + nab-paclitaxel	Phase1b study (NCT01633970) in the GP28328 multicenter: -Manageable safety and tolerability -Increase in activated proliferating CD8+ T cells in the peripheral blood promising anti-tumor activity and also better survival (156)
			High risk ductal TNBC +neoadjuvant chemotherapyNACT	NeoTRIPaPDL1 (NCT02620280) trial: -No significant increase of the rate of pCR (157).
Durvalumab			Germline BRCA-mutated metastatic breast cancer + Olaparib	Phase I/II trial (NCT02734004): Antitumor activity and safety similar (158)
			Early TNBC patients	Improved pathological complete response (159).
			Metastatic breast cancer patients	In the SAFIR02-BREAST IMMUNO trial(NCT02299999): -Disease remained stable after 6–8 rounds of chemotherapy - In the overall population, durvalumab did not improve progression-free survival or overall survival (160)
Tremelimumab	Humanized IgG2 monoclonal antibody, which inhibits tumor growth by preventing the interaction between CTLA-4 and B7 and thereby allowing T-cell activation (161)	CTLA-4	TNBC patients+ durvalumab	Immune activation by enhancing inducible co-stimulator activation on CD8 + and CD4 + T-cells (147) Better therapeutic responses stimulated local immune response and likely induce activated T cells to recognize cancer-specific antigens (161)
			mTNBC with brain metastasis and non-CNS measurable disease +brain radiotherapy (RT)	NCT02563925 clinical trial: -Modest clinical activity was observed in the HER2- efficacy cohort (162) - One patient with HER2+ disease experienced a durable partial response with evidence of peripheral T-cell activation (162)
Ipilimumab			Early-stage HER2−negative BC+ nivolumab + talimogene laherparepvec	T-VEC (NCT04185311): -One patient had a pathologic complete response, 3 patients had pathologic partial responses, 1 showed no significant response, and 1 had disease progression (163) -Biopsies demonstrated increased immune cell infiltration in samples from patients who responded to therapy (163)

PD-1, programmed cell death 1; PD-L1, programmed cell death-ligand; CTLA-4, cytotoxic T-Lymphocyte-associated-protein 4; IgG4, Immunoglobulin G4; IgG2, Immunoglobulin G2; ORR, objective response rate; BC, breast cancer; pCR, Pathological complete response; TNBC, triple negative breast cancer; mTNBC, metatstatic triple negative breast cancer; CR, complete response; ORR, objective response rate; HER2, human epidermal growth factor receptor 2; HR, hormone receptor; CR, complete response; PR, partial response; IgG1, Immunoglobulin G1; ADCC, antibody-dependent cell-mediated cytotoxicity; MBC, Metastatic breast cancer; RT, radiotherapy; CNS, central nervous system; CT, chemotherapy; NACT, neoadjuvant chemotherapy.

Bold values represent key immune checkpoint markers: PD-1 (Programmed Death-1), PD-L1 (Programmed Death-Ligand 1), and CTLA-4 (Cytotoxic T-Lymphocyte Associated Protein 4).

Pembrolizumab, a humanized IgG4 monoclonal antibody, has demonstrated high selectivity and affinity for PD-1 (**Table 1**), effectively inhibiting the interaction between PD-1 and its ligands, PD-L1 and PD-L2. In 2015, the U.S. Food and Drug Administration (FDA) approved pembrolizumab for the treatment of advanced melanoma. Subsequent clinical studies have validated its efficacy in various other cancer types, including gastric cancer, head and neck cancer, non-small cell lung cancer, and urothelial cancer (164).

A clinical trial involving 602 patients revealed that 64% exhibited a complete pathological response to pembrolizumab. Notably, patients who received pembrolizumab in conjunction with neoadjuvant therapy showed significantly improved responses compared to those who received a placebo with neoadjuvant therapy (128).

In the KEYNOTE-086 clinical study, which included 170 patients with metastatic triple-negative breast cancer (mTNBC) and a 68.1% PD-L1 positivity rate, pembrolizumab monotherapy was administered at a dose of 200 mg intravenously (IV) every three weeks for up to two years. The study reported an overall response rate of 5.7% in PD-L1 positive patients, with treatment-related adverse effects observed but no treatment-related deaths (165).

Avelumab, a fully human IgG1 monoclonal antibody, differs from other monoclonal antibodies in its selective inhibition of PD-1/PD-L1 interactions, while preserving PD-1/PD-L2 interactions. It features a functional crystallizable (Fc) fragment domain that engages Fc-γ receptors, thereby inducing antibody-dependent cellular cytotoxicity (ADCC) and complement-dependent cytotoxicity (CDC) in tumor cell lines during preclinical studies (152, 166). The FDA has approved avelumab for treating metastatic Merkel cell carcinoma (MCC) and locally advanced or metastatic urothelial carcinoma following progression after platinum-containing chemotherapy (167).

In the phase Ia/Ib JAVELIN study, 168 patients with metastatic breast cancer, including 58 with triple-negative breast cancer (TNBC), were treated with avelumab for durations ranging from 2 to 50 weeks and followed for 6 to 15 months. The overall response rate was 3.0%, with a 5.2% response rate in TNBC patients, suggesting that clinical responses may be heightened in PD-L1 expressing tumors. Avelumab demonstrated an acceptable safety profile in this cohort (168, 169).

Atezolizumab, a humanized, non-glycosylated monoclonal immunoglobulin G1 antibody, selectively targets and inhibits the interaction between PD-L1 and its receptor. This blockade indirectly lifts T-cell suppression, fostering an anti-tumor immune response. The efficacy of atezolizumab as a single agent was evaluated across various tumor types in a Phase I study. This cohort comprised 115 patients, of whom 33% exhibited PD-L1 positivity. The overall response rate among these patients was 10%, with a slightly higher rate of 13% observed in PD-L1 positive individuals (149, 170).

In a subsequent Phase Ib study (NCT01633970), 33 patients with either metastatic or locally recurrent Triple-Negative Breast Cancer (TNBC) were enrolled to assess the combined effect of atezolizumab and chemotherapy. Treatment protocol entailed administering 800mg of atezolizumab on the first and fifteenth days of each 28-day cycle, followed by nab-paclitaxel administration on days 1, 8, and 15 of the cycle. This regimen yielded an overall response rate of 39.4%, with superior outcomes noted in patients harboring PD-L1 positive tumors (156).

A Phase III clinical trial engaged a cohort of 902 patients, 90% of whom were diagnosed with metastatic disease. This study explored the efficacy of combining nab-paclitaxel with atezolizumab compared to nab-paclitaxel alone. Participants were evenly divided into two groups: one received a placebo plus nab-paclitaxel, and the other, atezolizumab plus nab-paclitaxel. Among these, 40.9% had PD-L1 positive tumors. Findings revealed that the median overall survival for patients treated with the placebo combination was 17.6 months, contrasted with 21.3 months for those receiving atezolizumab and nab-paclitaxel. Moreover, in the subset of patients with PD-L1 positive tumors, the overall survival spanned 15.5 months versus 25 months, respectively. These results conclusively demonstrate that combining atezolizumab with nab-paclitaxel significantly extends survival in affected patients (170).

## Novel therapeutic targets and perspectives

Recent research advances in breast cancer have identified many new therapeutic targets, offering promising approaches to treatment, particularly for aggressive subtypes such as TNBC. High-throughput approaches have identified critical molecules such as cyclin D-dependent kinases (CDK4/6 (171). Palbociclib is a novel, orally active pyridopyrimidine compound, which is highly selective reversible inhibitor of CDK4/6 (171). By inhibiting CDK4/6, palbociclib suppresses tumor cell entry into S-phase and reduces expression of the proliferation marker Ki67 (172). In preclinical studies, endocrine-resistant ER+ breast cancer cells were found to be extremely sensitive to palbociclib, with or even in the absence of antihormonal therapy (173).

Additionally, the PI3K/AKT/mTOR pathway is also being evaluated in clinical trials. It has been shown in various studies that the PI3K/AKT/mTOR pathway is activated in TNBC, due to loss of pTEN or INPP4B, or mutations in PIK3CA or AKT (174).

A clinical trial testing the effectiveness of the weekly combination of paclitaxel, doxorubicin and cyclophosphamide with the AKT inhibitor MK-2206 as a preoperative treatment for breast cancer revealed a significantly improved rate of complete tumor disappearance (40%) when compared with chemotherapy alone (22%) (175). In addition, several other molecular targets are under exploration to overcome the diversity of breast cancer. Such targets involve poly (ADP-ribose) polymerase (PARP). The BRCA mutations usually found in TNBC, are crucial in detecting and repairing DNA damage. Importantly when BRCA function is lost, it results in inducing cancer cell proliferation and progression (176). By using PARP inhibitors, the tumor cells undergo cell death, resulting in a highly effective therapeutic outcomes (177).

Circular RNA (CircRNA) has also been involved in tumorigenesis and drug resistance, offering promising targets for therapeutic approaches (178).

This highlights the importance of conducting further research into the molecular mechanisms of breast cancer in the interest of developing more efficient targeted therapies. The strategies currently used to deliver precision treatment are based essentially on the molecular subtyping of breast cancer. Future treatment concepts will focus more on individualizing therapy for each patient and tumor biology and early predictive markers will determine treatment. In addition, future research would have to focus on enhancing the safety, specificity and longevity of these therapies, as also on finding synergies between these new agents and their combination with already existing treatments.

## Conclusion

This article comprehensively reviews significant advancements in the field of breast cancer immunotherapy, underscoring the pivotal role of the immune system in both tumor suppression and progression. This increased understanding has catalyzed the development of targeted therapies and immunotherapeutic strategies.

Particularly, immune checkpoint inhibitors, including pembrolizumab, nivolumab, and atezolizumab, have yielded encouraging outcomes in clinical trials, showcasing enhanced response rates and prolonged survival. These therapies function by inhibiting pathways that limit the immune system’s ability to combat tumors, thereby intensifying the anti-tumor immune response.

Despite these advancements, challenges persist in counteracting the immune evasion mechanisms employed by breast cancer cells, such as PD-L1 overexpression and the recruitment of immunosuppressive cells into the TME. Current research is heavily focused on identifying predictive biomarkers and refining combination approaches with chemotherapy and other targeted treatments.

In conclusion, immunotherapy presents substantial promise, particularly for treating advanced and metastatic breast cancer. Ongoing research aimed at deepening our comprehension of the immune microenvironment is expected to facilitate the development of more efficacious and individualized therapies, ultimately enhancing patient outcomes in breast cancer treatment. Overall, especially in advanced and metastatic breast cancer, immunotherapy holds great promise. Continued research and advances in understanding the immune microenvironment will contribute to the development of more effective and personalized therapies, ultimately improving outcomes for breast cancer patients.

## References

[1] Siegel RL Miller KD Jemal A. Cancer statistics, 2018. CA Cancer J Clin. (2018) 68:7–30. doi: 10.3322/caac.21442 29313949

[2] Ferlay J Colombet M Soerjomataram I Parkin DM Piñeros M Znaor A. Cancer statistics for the year 2020: An overview. Int J Cancer. (2021) 149:778–789. doi: 10.1002/ijc.33588 33818764

[3] Ginsburg O Bray F Coleman MP Vanderpuye V Eniu A Kotha SR. The global burden of women’s cancers: a grand challenge in global health. Lancet. (2017) 389:847–60. doi: 10.1016/S0140-6736(16)31392-7 PMC6191029 27814965

[4] Perou CM Sørlie T Eisen MB van de Rijn M Jeffrey SS Rees CA. Molecular portraits of human breast tumours. Nature. (2000) 406:747–52. doi: 10.1038/35021093 10963602

[5] Sørlie T Perou CM Tibshirani R Aas T Geisler S Johnsen H. Gene expression patterns of breast carcinomas distinguish tumor subclasses with clinical implications. Proc Natl Acad Sci U S A. (2001) 98:10869–74. doi: 10.1073/pnas.191367098 PMC58566 11553815

[6] Gradishar WJ Anderson BO Balassanian R Blair SL Burstein HJ Cyr A. Breast cancer, version 4.2017, NCCN clinical practice guidelines in oncology. J Natl Compr Canc Netw. (2018) 16:310–20. doi: 10.6004/jnccn.2018.0012 29523670

[7] Harbeck N Penault-Llorca F Cortes J Gnant M Houssami N Poortmans P. Breast cancer. Nat Rev Dis Primers. (2019) 5:1–31. doi: 10.1038/s41572-019-0111-2 31548545

[8] Angelico G Broggi G Tinnirello G Puzzo L Vecchio GM Salvatorelli L. Tumor infiltrating lymphocytes (TILS) and PD-L1 expression in breast cancer: A review of current evidence and prognostic implications from pathologist’s perspective. Cancers. (2023) 15:4479. doi: 10.3390/cancers15184479 37760449 PMC10526828

[9] A review of the importance of immune responses in luminal B breast cancer - PMC. Available online at: https://www.ncbi.nlm.nih.gov/pmc/articles/PMC5384410/ (Accessed August 12, 2024).

[10] Hammerl D Massink MPG Smid M van Deurzen CHM Meijers-Heijboer HEJ Waisfisz Q. Clonality, antigen recognition, and suppression of CD8+ T cells differentially affect prognosis of breast cancer subtypes. Clin Cancer Res. (2020) 26:505–17. doi: 10.1158/1078-0432.CCR-19-0285 31649042

[11] Moragon S Hernando C Martinez-Martinez MT Tapia M Ortega-Morillo B Lluch A. Immunological landscape of HER-2 positive breast cancer. Cancers (Basel). (2022) 14:3167. doi: 10.3390/cancers14133167 35804943 PMC9265068

[12] Savas P Salgado R Denkert C Sotiriou C Darcy PK Smyth MJ. Clinical relevance of host immunity in breast cancer: from TILs to the clinic. Nat Rev Clin Oncol. (2016) 13:228–41. doi: 10.1038/nrclinonc.2015.215 26667975

[13] Emens LA. Breast cancer immunotherapy: facts and hopes. Clin Cancer Res. (2018) 24:511–20. doi: 10.1158/1078-0432.CCR-16-3001 PMC5796849 28801472

[14] Goldhirsch A Wood WC Coates AS Gelber RD Thürlimann B Senn H-J. Strategies for subtypes—dealing with the diversity of breast cancer: highlights of the St Gallen International Expert Consensus on the Primary Therapy of Early Breast Cancer 2011. Ann Oncol. (2011) 22:1736–47. doi: 10.1093/annonc/mdr304 PMC3144634 21709140

[15] Derouane F van Marcke C Berlière M Gerday A Fellah L Leconte I. Predictive biomarkers of response to neoadjuvant chemotherapy in breast cancer: current and future perspectives for precision medicine. Cancers. (2022) 14:3876. doi: 10.3390/cancers14163876 36010869 PMC9405974

[16] Molecular classification of breast cancer: A retrospective cohort study - ScienceDirect. Available online at: https://www.sciencedirect.com/science/article/pii/S204908011930192Xbib9 (Accessed June 4, 2024).

[17] Fahad Ullah M. Breast cancer: current perspectives on the disease status. In: Ahmad A, editor. Breast Cancer Metastasis and Drug Resistance: Challenges and Progress. Advances in Experimental Medicine and Biology. Springer International Publishing, Cham (2019). p. 51–64. doi: 10.1007/978-3-030-20301-6_4 31456179

[18] Shida D Kanemitsu Y Hamaguchi T Shimada Y. Introducing the eighth edition of the tumor-node-metastasis classification as relevant to colorectal cancer, anal cancer and appendiceal cancer: a comparison study with the seventh edition of the tumor-node-metastasis and the Japanese Classification of Colorectal, Appendiceal, and Anal Carcinoma. Japanese J Clin Oncol. (2019) 49:321–8. doi: 10.1093/jjco/hyy198 30608547

[19] Łukasiewicz S Czeczelewski M Forma A Baj J Sitarz R Stanisławek A. Breast cancer—Epidemiology, risk factors, classification, prognostic markers, and current treatment strategies—An updated review. Cancers (Basel). (2021) 13:4287. doi: 10.3390/cancers13174287 34503097 PMC8428369

[20] Spranger S Sivan A Corrales L Gajewski TF. Tumor and host factors controlling antitumor immunity and efficacy of cancer immunotherapy. Adv Immunol. (2016) 130:75–93. doi: 10.1016/bs.ai.2015.12.003 26923000 PMC4864964

[21] Ye Y Xu C Chen F Liu Q Cheng N. Targeting innate immunity in breast cancer therapy: A narrative review. Front Immunol. (2021) 12:771201. doi: 10.3389/fimmu.2021.771201 34899721 PMC8656691

[22] Smyth MJ Godfrey DI Trapani JA. A fresh look at tumor immunosurveillance and immunotherapy. Nat Immunol. (2001) 2:293–9. doi: 10.1038/86297 11276199

[23] Guan P Guo Z Xu S Lu H Wang L Gu Z. Molecularly imprinted nanobeacons redirect innate immune killing towards triple negative breast cancer. Angewandte Chemie Int Edition. (2023) 62:e202301202. doi: 10.1002/anie.202301202 36814079

[24] Demaria O Cornen S Daëron M Morel Y Medzhitov R Vivier E. Harnessing innate immunity in cancer therapy. Nature. (2019) 574:45–56. doi: 10.1038/s41586-019-1593-5 31578484

[25] Hanahan D Coussens LM. Accessories to the crime: functions of cells recruited to the tumor microenvironment. Cancer Cell. (2012) 21:309–22. doi: 10.1016/j.ccr.2012.02.022 22439926

[26] Zhang Y Wang Q Yang W-K Wang Y-S Zhou Q Lin J. Development of an immune-related prognostic biomarker for triple-negative breast cancer. Ann Med. (2022) 54:1212–20. doi: 10.1080/07853890.2022.2067894 PMC9068007 35481432

[27] Hourani T Holden JA Li W Lenzo JC Hadjigol S O’Brien-Simpson NM. Tumor associated macrophages: origin, recruitment, phenotypic diversity, and targeting. Front Oncol. (2021) 11:788365. doi: 10.3389/fonc.2021.788365 34988021 PMC8722774

[28] Mantovani A Sica A Sozzani S Allavena P Vecchi A Locati M. The chemokine system in diverse forms of macrophage activation and polarization. Trends Immunol. (2004) 25:677–86. doi: 10.1016/j.it.2004.09.015 15530839

[29] Liu Q Hodge J Wang J Wang Y Wang L Singh UP. Emodin reduces Breast Cancer Lung Metastasis by suppressing Macrophage-induced Breast Cancer Cell Epithelial-mesenchymal transition and Cancer Stem Cell formation. Theranostics. (2020) 10:8365–81. doi: 10.7150/thno.45395 PMC7381725 32724475

[30] Pollard JW. Trophic macrophages in development and disease. Nat Rev Immunol. (2009) 9:259–70. doi: 10.1038/nri2528 PMC3648866 19282852

[31] Zhang S-C Hu Z-Q Long J-H Zhu G-M Wang Y Jia Y. Clinical implications of tumor-infiltrating immune cells in breast cancer. J Cancer. (2019) 10:6175–84. doi: 10.7150/jca.35901 PMC6856577 31762828

[32] Bense RD Sotiriou C Piccart-Gebhart MJ Haanen JBAG van Vugt MATM de Vries EGE. Relevance of tumor-infiltrating immune cell composition and functionality for disease outcome in breast cancer. JNCI J Natl Cancer Inst. (2017) 109:djw192. doi: 10.1093/jnci/djw192 27737921 PMC6284248

[33] Barbera-Guillem E Nyhus JK Wolford CC Friece CR Sampsel JW. Vascular endothelial growth factor secretion by tumor-infiltrating macrophages essentially supports tumor angiogenesis, and IgG immune complexes potentiate the process. Cancer Res. (2002) 62:7042–9. 12460925

[34] Tan DQ Zhang L Ohba K Ye M Ichiyama K Yamamoto N. Macrophage response to oncolytic paramyxoviruses potentiates virus-mediated tumor cell killing. Eur J Immunol. (2016) 46:919–28. doi: 10.1002/eji.201545915 26763072

[35] Parkes EE Savage KI Lioe T Boyd C Halliday S Walker SM. Activation of a cGAS-STING-mediated immune response predicts response to neoadjuvant chemotherapy in early breast cancer. Br J Cancer. (2022) 126:247–58. doi: 10.1038/s41416-021-01599-0 PMC8770594 34728791

[36] Lin H-J Liu Y Lofland D Lin J. Breast cancer tumor microenvironment and molecular aberrations hijack tumoricidal immunity. Cancers. (2022) 14:285. doi: 10.3390/cancers14020285 35053449 PMC8774102

[37] Davra V Kumar S Geng K Calianese D Mehta D Gadiyar V. Axl and Mertk receptors cooperate to promote breast cancer progression by combined oncogenic signaling and evasion of host antitumor immunity. Cancer Res. (2021) 81:698–712. doi: 10.1158/0008-5472.CAN-20-2066 33239426 PMC9999365

[38] Qian B-Z Pollard JW. Macrophage diversity enhances tumor progression and metastasis. Cell. (2010) 141:39–51. doi: 10.1016/j.cell.2010.03.014 20371344 PMC4994190

[39] Liu Y Cao X. The origin and function of tumor-associated macrophages. Cell Mol Immunol. (2015) 12:1–4. doi: 10.1038/cmi.2014.83 25220733 PMC4654376

[40] Noy R Pollard JW. Tumor-associated macrophages: from mechanisms to therapy. Immunity. (2014) 41:49–61. doi: 10.1016/j.immuni.2014.06.010 25035953 PMC4137410

[41] Franklin RA Liao W Sarkar A Kim MV Bivona MR Liu K. The cellular and molecular origin of tumor-associated macrophages. Science. (2014) 344:921–5. doi: 10.1126/science.1252510 PMC4204732 24812208

[42] Cheng N Bei Y Song Y Zhang W Xu L Zhang W. B7-H3 augments the pro-angiogenic function of tumor-associated macrophages and acts as a novel adjuvant target for triple-negative breast cancer therapy. Biochem Pharmacol. (2021) 183:114298. doi: 10.1016/j.bcp.2020.114298 33153969

[43] Wang N Liu W Zheng Y Wang S Yang B Li M. CXCL1 derived from tumor-associated macrophages promotes breast cancer metastasis via activating NF-κB/SOX4 signaling. Cell Death Dis. (2018) 9:880. doi: 10.1038/s41419-018-0876-3 30158589 PMC6115425

[44] Marquez-Palencia M Malladi S. Abstract PD4-07: Targeting latent residual HER2+ breast cancer brain metastatic cells. Cancer Res. (2022) 82:PD4–07. doi: 10.1158/1538-7445.SABCS21-PD4-07

[45] Rafii S Kandoussi S Ghouzlani A Naji O Reddy KP Ullah Sadiqi R. Deciphering immune microenvironment and cell evasion mechanisms in human gliomas. Front Oncol. (2023) 13:1135430. doi: 10.3389/fonc.2023.1135430 37274252 PMC10235598

[46] Hanamura T Kitano S Kagamu H Yamashita M Terao M Tsuda B. Immunological profiles of the breast cancer microenvironment represented by tumor-infiltrating lymphocytes and PD-L1 expression. Sci Rep. (2022) 12:8098. doi: 10.1038/s41598-022-11578-x 35577913 PMC9110375

[47] Wei H-C. Mathematical modeling of tumor growth and treatment: Triple negative breast cancer. Mathematics Comput Simulation. (2023) 204:645–59. doi: 10.1016/j.matcom.2022.09.005

[48] Chan IS Knútsdóttir H Ramakrishnan G Padmanaban V Warrier M Ramirez JC. Cancer cells educate natural killer cells to a metastasis-promoting cell state. J Cell Biol. (2020) 219:e202001134. doi: 10.1083/jcb.202001134 32645139 PMC7480097

[49] Briukhovetska D Suarez-Gosalvez J Voigt C Markota A Giannou AD Schübel M. T cell-derived interleukin-22 drives the expression of CD155 by cancer cells to suppress NK cell function and promote metastasis. Immunity. (2023) 56:143–61.e11. doi: 10.1016/j.immuni.2022.12.010 36630913 PMC9839367

[50] Konjević GM Vuletić AM Mirjačić Martinović KM Larsen AK Jurišić VB. The role of cytokines in the regulation of NK cells in the tumor environment. Cytokine. (2019) 117:30–40. doi: 10.1016/j.cyto.2019.02.001 30784898

[51] Alter G Malenfant JM Altfeld M. CD107a as a functional marker for the identification of natural killer cell activity. J Immunol Methods. (2004) 294:15–22. doi: 10.1016/j.jim.2004.08.008 15604012

[52] The pharmalogical reactivation of p53 function improves breast tumor cell lysis by granzyme B and NK cells through induction of autophagy - PubMed. Available online at: https://pubmed.ncbi.nlm.nih.gov/31541080/ (Accessed March 28, 2023). 10.1038/s41419-019-1950-1 PMC6754511 31541080

[53] The overexpression and clinical significance of AP1S1 in breast cancer - PMC. Available online at: https://www.ncbi.nlm.nih.gov/pmc/articles/PMC9021008/ (Accessed March 28, 2023).

[54] Zhou W Yu M Mao X Pan H Tang X Wang J. Landscape of the peripheral immune response induced by local microwave ablation in patients with breast cancer. Adv Sci (Weinh). (2022) 9:e2200033. doi: 10.1002/advs.202200033 35403824 PMC9189675

[55] Benci JL Xu B Qiu Y Wu TJ Dada H Twyman-Saint Victor C. Tumor interferon signaling regulates a multigenic resistance program to immune checkpoint blockade. Cell. (2016) 167:1540–54.e12. doi: 10.1016/j.cell.2016.11.022 27912061 PMC5385895

[56] Nicolini A Carpi A. Immune manipulation of advanced breast cancer: an interpretative model of the relationship between immune system and tumor cell biology. Med Res Rev. (2009) 29:436–71. doi: 10.1002/med.20143 19105214

[57] Marrufo AM Mathew SO Chaudhary P Malaer JD Vishwanatha JK Mathew PA. Blocking LLT1 (CLEC2D, OCIL)-NKRP1A (CD161) interaction enhances natural killer cell-mediated lysis of triple-negative breast cancer cells. Am J Cancer Res. (2018) 8:1050–63. PMC6048397 30034942

[58] Park IH Yang HN Lee KJ Kim T-S Lee ES Jung S-Y. Tumor-derived IL-18 induces PD-1 expression on immunosuppressive NK cells in triple-negative breast cancer. Oncotarget. (2017) 8:32722–30. doi: 10.18632/oncotarget.16281 PMC5464822 28415798

[59] Breunig C Pahl J Küblbeck M Miller M Antonelli D Erdem N. MicroRNA-519a-3p mediates apoptosis resistance in breast cancer cells and their escape from recognition by natural killer cells. Cell Death Dis. (2017) 8:e2973. doi: 10.1038/cddis.2017.364 28771222 PMC5596553

[60] Feng H Dong Y Wu J Qiao Y Zhu G Jin H. Epirubicin pretreatment enhances NK cell-mediated cytotoxicity against breast cancer cells. vitro Am J Transl Res. (2016) 8:473–84. PMC4846897 27158340

[61] Beitsch P Lotzová E Hortobagyi G Pollock R. Natural immunity in breast cancer patients during neoadjuvant chemotherapy and after surgery. Surg Oncol. (1994) 3:211–9. doi: 10.1016/0960-7404(94)90036-1 7834112

[62] Sewell HF Halbert CF Robins RA Galvin A Chan S Blamey RW. Chemotherapy-induced differential changes in lymphocyte subsets and natural-killer-cell function in patients with advanced breast cancer. Int J Cancer. (1993) 55:735–8. doi: 10.1002/ijc.2910550506 7902339

[63] Solomayer E-F Feuerer M Bai L Umansky V Beckhove P Meyberg GC. Influence of adjuvant hormone therapy and chemotherapy on the immune system analysed in the bone marrow of patients with breast cancer1. Clin Cancer Res. (2003) 9:174–80. 12538466

[64] Roberti MP Juliá EP Rocca YS Amat M Bravo AI Loza J. Overexpression of CD85j in TNBC patients inhibits Cetuximab-mediated NK-cell ADCC but can be restored with CD85j functional blockade. Eur J Immunol. (2015) 45:1560–9. doi: 10.1002/eji.201445353 25726929

[65] Neo SY Yang Y Record J Ma R Chen X Chen Z. CD73 immune checkpoint defines regulatory NK cells within the tumor microenvironment. J Clin Invest. (2020) 130:1185–98. doi: 10.1172/jci128895 PMC7269592 31770109

[66] Petsikas D Mohamed F Ricci M Symes J Guerraty A. Adenosine enhances left ventricular flow during 24-hour hypothermic perfusion of isolated cardiac allografts. J Heart Transplant. (1990) 9:543–7. 2231093

[67] Full article: Antibodies to watch in 2020 (Accessed March 28, 2023).

[68] Bang YJ Giaccone G Im SA Oh DY Bauer TM Nordstrom JL. First-in-human phase 1 study of margetuximab (MGAH22), an Fc-modified chimeric monoclonal antibody, in patients with HER2-positive advanced solid tumors. Ann Oncol. (2017) 28:855–61. doi: 10.1093/annonc/mdx002 PMC6246722 28119295

[69] Mazloumi Z Rafat A Dizaji Asl K Nozad Charoudeh H. A combination of telomerase inhibition and NK cell therapy increased breast cancer cell line apoptosis. Biochem Biophys Res Commun. (2023) 640:50–5. doi: 10.1016/j.bbrc.2022.11.090 36502631

[70] Chen Q He L Li X Xu L Chen T. Ruthenium complexes boost NK cell immunotherapy via sensitizing triple-negative breast cancer and shaping immuno-microenvironment. Biomaterials. (2022) 281:121371. doi: 10.1016/j.biomaterials.2022.121371 35063740

[71] Mattiuz R Brousse C Ambrosini M Cancel J Bessou G Mussard J. Type 1 conventional dendritic cells and interferons are required for spontaneous CD4+ and CD8+ T-cell protective responses to breast cancer. Clin Transl Immunol. (2021) 10:e1305. doi: 10.1002/cti2.1305 PMC8279130 34277006

[72] Hubert M Gobbini E Couillault C Manh T-PV Doffin A-C Berthet J. IFN-III is selectively produced by cDC1 and predicts good clinical outcome in breast cancer. Sci Immunol. (2020) 5:eaav3942. doi: 10.1126/sciimmunol.aav3942 32303573

[73] Szpor J Streb J Glajcar A Frączek P Winiarska A Tyrak KE. Dendritic cells are associated with prognosis and survival in breast cancer. Diagnostics (Basel). (2021) 11:702. doi: 10.3390/diagnostics11040702 33919875 PMC8070803

[74] Reichert TE Scheuer C Day R Wagner W Whiteside TL. The number of intratumoral dendritic cells and zeta-chain expression in T cells as prognostic and survival biomarkers in patients with oral carcinoma. Cancer. (2001) 91:2136–47. doi: 10.1002/1097-0142(20010601)91:11<2136::AID-CNCR1242>3.0.CO;2-Q 11391595

[75] La Rocca G Anzalone R Corrao S Magno F Rappa F Marasà S. CD1a down-regulation in primary invasive ductal breast carcinoma may predict regional lymph node invasion and patient outcome. Histopathology. (2008) 52:203–12. doi: 10.1111/j.1365-2559.2007.02919.x 18184269

[76] Sisirak V Faget J Gobert M Goutagny N Vey N Treilleux I. Impaired IFN-α production by plasmacytoid dendritic cells favors regulatory T-cell expansion that may contribute to breast cancer progression. Cancer Res. (2012) 72:5188–97. doi: 10.1158/0008-5472.CAN-11-3468 22836755

[77] Treilleux I Blay J-Y Bendriss-Vermare N Ray-Coquard I Bachelot T Guastalla J-P. Dendritic cell infiltration and prognosis of early stage breast cancer. Clin Cancer Res. (2004) 10:7466–74. doi: 10.1158/1078-0432.CCR-04-0684 15569976

[78] Li J Zhao M Liang W Wu S Wang Z Wang D. Codelivery of Shikonin and siTGF-β for enhanced triple negative breast cancer chemo-immunotherapy. J Control Release. (2022) 342:308–20. doi: 10.1016/j.jconrel.2022.01.015 35031387

[79] Acikgoz E Duzagac F Guven U Yigitturk G Kose T Oktem G. Double hit” strategy: Removal of sialic acid from the dendritic cell surface and loading with CD44+/CD24-/low cell lysate inhibits tumor growth and metastasis by targeting breast cancer stem cells. Int Immunopharmacol. (2022) 107:108684. doi: 10.1016/j.intimp.2022.108684 35272171

[80] Kass R Agha J Bellone S Palmieri M Canè S Bignotti E. *In vitro* induction of tumor-specific HLA class I-restricted CD8+ cytotoxic T lymphocytes from patients with locally advanced breast cancer by tumor antigen-pulsed autologous dendritic cells. J Surg Res. (2003) 112:189–97. doi: 10.1016/s0022-4804(03)00147-1 12888337

[81] Bernal-Estévez DA Ortíz Barbosa MA Ortíz-Montero P Cifuentes C Sánchez R Parra-López CA. Autologous dendritic cells in combination with chemotherapy restore responsiveness of T cells in breast cancer patients: A single-arm phase I/II trial. Front Immunol. (2021) 12:669965. doi: 10.3389/fimmu.2021.669965 34489928 PMC8417880

[82] Zhang J Pan S Jian C Hao L Dong J Sun Q. Immunostimulatory properties of chemotherapy in breast cancer: from immunogenic modulation mechanisms to clinical practice. Front Immunol. (2022) 12:819405. doi: 10.3389/fimmu.2021.819405 35069604 PMC8766762

[83] Gatti-Mays ME Balko JM Gameiro SR Bear HD Prabhakaran S Fukui J. If we build it they will come: targeting the immune response to breast cancer. NPJ Breast Cancer. (2019) 5:1–13. doi: 10.1038/s41523-019-0133-7 31700993 PMC6820540

[84] Carbone DP Gandara DR Antonia SJ Zielinski C Paz-Ares L. Non–small cell lung cancer: role of the immune system and potential for immunotherapy. J Thorac Oncol. (2015) 10:974–84. doi: 10.1097/JTO.0000000000000551 PMC4618296 26134219

[85] Ciarka A Piątek M Pęksa R Kunc M Senkus E. Tumor-infiltrating lymphocytes (TILs) in breast cancer: prognostic and predictive significance across molecular subtypes. Biomedicines. (2024) 12:763. doi: 10.3390/biomedicines12040763 38672117 PMC11048219

[86] Chraa D Naim A Olive D Badou A. T lymphocyte subsets in cancer immunity: Friends or foes. J Leukoc Biol. (2019) 105:243–55. doi: 10.1002/JLB.MR0318-097R 30387907

[87] Adaptive immunity in the liver - PubMed. Available online at: https://pubmed.ncbi.nlm.nih.gov/26996069/ (Accessed March 28, 2023).

[88] Matsushita H Vesely MD Koboldt DC Rickert CG Uppaluri R Magrini VJ. Cancer exome analysis reveals a T-cell-dependent mechanism of cancer immunoediting. Nature. (2012) 482:400–4. doi: 10.1038/nature10755 PMC3874809 22318521

[89] Ghouzlani A Rafii S Karkouri M Lakhdar A Badou A. The promising IgSF11 immune checkpoint is highly expressed in advanced human gliomas and associates to poor prognosis. Front Oncol. (2021) 10:608609. doi: 10.3389/fonc.2020.608609 33604291 PMC7884863

[90] Ghouzlani A Lakhdar A Rafii S Karkouri M Badou A. The immune checkpoint VISTA exhibits high expression levels in human gliomas and associates with a poor prognosis. Sci Rep. (2021) 11:21504. doi: 10.1038/s41598-021-00835-0 34728682 PMC8563991

[91] Sato E Olson SH Ahn J Bundy B Nishikawa H Qian F. Intraepithelial CD8+ tumor-infiltrating lymphocytes and a high CD8+/regulatory T cell ratio are associated with favorable prognosis in ovarian cancer. Proc Natl Acad Sci. (2005) 102:18538–43. doi: 10.1073/pnas.0509182102 PMC1311741 16344461

[92] Fridman WH Pagès F Sautès-Fridman C Galon J. The immune contexture in human tumours: impact on clinical outcome. Nat Rev Cancer. (2012) 12:298–306. doi: 10.1038/nrc3245 22419253

[93] Sun Y-P Ke Y-L Li X. Prognostic value of CD8+ tumor-infiltrating T cells in patients with breast cancer: A systematic review and meta-analysis. Oncol Lett. (2022) 25:39. doi: 10.3892/ol.2022.13625 36589661 PMC9773320

[94] Philip M Schietinger A. CD8+ T cell differentiation and dysfunction in cancer. Nat Rev Immunol. (2022) 22:209–23. doi: 10.1038/s41577-021-00574-3 PMC9792152 34253904

[95] Nelson MA Ngamcherdtrakul W Luoh S-W Yantasee W. Prognostic and therapeutic role of tumor-infiltrating lymphocyte subtypes in breast cancer. Cancer Metastasis Rev. (2021) 40:519–36. doi: 10.1007/s10555-021-09968-0 PMC8424653 33963482

[96] Ngamcherdtrakul W Yantasee W. siRNA therapeutics for breast cancer: recent efforts in targeting metastasis, drug resistance, and immune evasion. Transl Res. (2019) 214:105–20. doi: 10.1016/j.trsl.2019.08.005 PMC6848785 31487500

[97] Buisseret L Garaud S de Wind A Van den Eynden G Boisson A Solinas C. Tumor-infiltrating lymphocyte composition, organization and PD-1/ PD-L1 expression are linked in breast cancer. OncoImmunology. (2017) 6:e1257452. doi: 10.1080/2162402X.2016.1257452 28197375 PMC5283629

[98] Formenti SC Hawtin RE Dixit N Evensen E Lee P Goldberg JD. Baseline T cell dysfunction by single cell network profiling in metastatic breast cancer patients. J Immunother Cancer. (2019) 7:177. doi: 10.1186/s40425-019-0633-x 31296256 PMC6624899

[99] Park H Lim Y Ko ES Cho H-H Lee JE Han B-K. Radiomics signature on magnetic resonance imaging: association with disease-free survival in patients with invasive breast cancer. Clin Cancer Res. (2018) 24:4705–14. doi: 10.1158/1078-0432.CCR-17-3783 29914892

[100] Salisbury T Abozina A Zhang C Mao E Banyi N Leo J. Histological subtype is associated with PD-L1 expression and CD8+ T-cell infiltrates in triple-negative breast carcinoma. Ann Diagn Pathol. (2022) 57:151901. doi: 10.1016/j.anndiagpath.2022.151901 35091156

[101] Nanda R Chow LQM Dees EC Berger R Gupta S Geva R. Pembrolizumab in patients with advanced triple-negative breast cancer: phase Ib KEYNOTE-012 study. J Clin Oncol. (2016) 34:2460–7. doi: 10.1200/JCO.2015.64.8931 PMC6816000 27138582

[102] Human breast tumor-infiltrating CD8+ T cells retain polyfunctionality despite PD-1 expression | Nature Communications. Available online at: https://www.nature.com/articles/s41467-018-06653-9 (Accessed March 28, 2023). 10.1038/s41467-018-06653-9 PMC6191461 30327458

[103] Revisiting the role of CD4+ T cells in cancer immunotherapy—new insights into old paradigms | Cancer Gene Therapy. Available online at: https://www.nature.com/articles/s41417-020-0183-x (Accessed March 28, 2023). 10.1038/s41417-020-0183-x PMC7886651 32457487

[104] Ahrends T Borst J. The opposing roles of CD4+ T cells in anti-tumour immunity. Immunology. (2018) 154:582–92. doi: 10.1111/imm.12941 PMC6050207 29700809

[105] Kennedy R Celis E. Multiple roles for CD4+ T cells in anti-tumor immune responses. Immunol Rev. (2008) 222:129–44. doi: 10.1111/j.1600-065X.2008.00616.x 18363998

[106] Oh DY Kwek SS Raju SS Li T McCarthy E Chow E. Intratumoral CD4+ T cells mediate anti-tumor cytotoxicity in human bladder cancer. Cell. (2020) 181:1612–25.e13. doi: 10.1016/j.cell.2020.05.017 32497499 PMC7321885

[107] Ben Khelil M Godet Y Abdeljaoued S Borg C Adotévi O Loyon R. Harnessing antitumor CD4+ T cells for cancer immunotherapy. Cancers. (2022) 14:260. doi: 10.3390/cancers14010260 35008422 PMC8750687

[108] Bevan MJ. Helping the CD8(+) T-cell response. Nat Rev Immunol. (2004) 4:595–602. doi: 10.1038/nri1413 15286726

[109] Huang Y Ma C Zhang Q Ye J Wang F Zhang Y. CD4+ and CD8+ T cells have opposing roles in breast cancer progression and outcome. Oncotarget. (2015) 6:17462–78. doi: 10.18632/oncotarget.v6i19 PMC4627321 25968569

[110] Azizi E Carr AJ Plitas G Cornish AE Konopacki C Prabhakaran S. Single-cell map of diverse immune phenotypes in the breast tumor microenvironment. Cell. (2018) 174:1293–308.e36. doi: 10.1016/j.cell.2018.05.060 29961579 PMC6348010

[111] CD4 T-cell immune stimulation of HER2 + breast cancer cells alters response to trastuzumab in *vitro* | Cancer Cell International | Full Text (Accessed March 28, 2023). 10.1186/s12935-020-01625-w PMC7654187 33292267

[112] CD4+ T helper 2 cells suppress breast cancer by inducing terminal differentiation - PMC. Available online at: https://www.ncbi.nlm.nih.gov/pmc/articles/PMC9170526/ (Accessed March 28, 2023). 10.1084/jem.20201963 PMC9170526 35657353

[113] Lhuillier C Rudqvist N-P Yamazaki T Zhang T Charpentier M Galluzzi L. Radiotherapy-exposed CD8+ and CD4+ neoantigens enhance tumor control. J Clin Invest. (2021) 131:e138740. doi: 10.1172/JCI138740 33476307 PMC7919731

[114] De Silva NS Klein U. Dynamics of B cells in germinal centres. Nat Rev Immunol. (2015) 15:137–48. doi: 10.1038/nri3804 PMC4399774 25656706

[115] Roles of the immune system in cancer: from tumor initiation to metastatic progression. Available online at: https://genesdev.cshlp.org/content/32/19-20/1267.full (Accessed March 28, 2023). 10.1101/gad.314617.118 PMC6169832 30275043

[116] Downs-Canner SM Meier J Vincent BG Serody JS. B cell function in the tumor microenvironment. Annu Rev Immunol. (2022) 40:169–93. doi: 10.1146/annurev-immunol-101220-015603 35044794

[117] Liu C Richard K Wiggins M Zhu X Conrad DH Song W. CD23 can negatively regulate B-cell receptor signaling. Sci Rep. (2016) 6:25629. doi: 10.1038/srep25629 27181049 PMC4867583

[118] Kuroda H Jamiyan T Yamaguchi R Kakumoto A Abe A Harada O. Prognostic value of tumor-infiltrating B lymphocytes and plasma cells in triple-negative breast cancer. Breast Cancer. (2021) 28:904–14. doi: 10.1007/s12282-021-01227-y PMC8213582 33629216

[119] Schnellhardt S Erber R Büttner-Herold M Rosahl M-C Ott OJ Strnad V. Tumour-infiltrating inflammatory cells in early breast cancer: an underrated prognostic and predictive factor? Int J Mol Sci. (2020) 21:8238. doi: 10.3390/ijms21218238 33153211 PMC7663093

[120] Li M Quintana A Alberts E Hung MS Boulat V Ripoll MM. B cells in breast cancer pathology. Cancers. (2023) 15:1517. doi: 10.3390/cancers15051517 36900307 PMC10000926

[121] Wouters MCA Nelson BH. Prognostic significance of tumor-infiltrating B cells and plasma cells in human cancer. Clin Cancer Res. (2018) 24:6125–35. doi: 10.1158/1078-0432.CCR-18-1481 30049748

[122] B cells and T follicular helper cells mediate response to checkpoint inhibitors in high mutation burden mouse models of breast cancer - pubMed. Available online at: https://pubmed.ncbi.nlm.nih.gov/31730857/ (Accessed March 28, 2023). 10.1016/j.cell.2019.10.028 PMC6911685 31730857

[123] Olkhanud PB Damdinsuren B Bodogai M Gress RE Sen R Wejksza K. Tumor-evoked regulatory B cells promote breast cancer metastasis by converting resting CD4^+^ T cells to T-regulatory cells. Cancer Res. (2011) 71:3505–15. doi: 10.1158/0008-5472.CAN-10-4316 PMC3096701 21444674

[124] Sarvaria A Madrigal JA Saudemont A. B cell regulation in cancer and anti-tumor immunity. Cell Mol Immunol. (2017) 14:662–74. doi: 10.1038/cmi.2017.35 PMC5549607 28626234

[125] Ishigami E Sakakibara M Sakakibara J Masuda T Fujimoto H Hayama S. Coexistence of regulatory B cells and regulatory T cells in tumor-infiltrating lymphocyte aggregates is a prognostic factor in patients with breast cancer. Breast Cancer. (2019) 26:180–9. doi: 10.1007/s12282-018-0910-4 30244409

[126] Liu M Wei F Wang J Yu W Shen M Liu T. Myeloid-derived suppressor cells regulate the immunosuppressive functions of PD-1-PD-L1+ Bregs through PD-L1/PI3K/AKT/NF-κB axis in breast cancer. Cell Death Dis. (2021) 12:465. doi: 10.1038/s41419-021-03745-1 33967272 PMC8107179

[127] Guan H Wan Y Lan J Wang Q Wang Z Li Y. PD-L1 is a critical mediator of regulatory B cells and T cells in invasive breast cancer. Sci Rep. (2016) 6:35651. doi: 10.1038/srep35651 27762298 PMC5071845

[128] Schmid P Cortes J Pusztai L McArthur H Kümmel S Bergh J. Pembrolizumab for early triple-negative breast cancer. N Engl J Med. (2020) 382:810–21. doi: 10.1056/NEJMoa1910549 32101663

[129] Conejo-Garcia JR Biswas S Chaurio R Rodriguez PC. Neglected no more: B cell-mediated anti-tumor immunity. Semin Immunol. (2023) 65:101707. doi: 10.1016/j.smim.2022.101707 36527759 PMC10123518

[130] Regulatory T lymphocyte infiltration in metastatic breast cancer—an independent prognostic factor that changes with tumor progression | Breast Cancer Research | Full Text (Accessed March 28, 2023). 10.1186/s13058-021-01403-0 PMC7893927 33602289

[131] Liu S Foulkes WD Leung S Gao D Lau S Kos Z. Prognostic significance of FOXP3+ tumor-infiltrating lymphocytes in breast cancer depends on estrogen receptor and human epidermal growth factor receptor-2 expression status and concurrent cytotoxic T-cell infiltration. Breast Cancer Res. (2014) 16:432. doi: 10.1186/s13058-014-0432-8 25193543 PMC4303113

[132] Plitas G Konopacki C Wu K Bos PD Morrow M Putintseva EV. Regulatory T cells exhibit distinct features in human breast cancer. Immunity. (2016) 45:1122–34. doi: 10.1016/j.immuni.2016.10.032 PMC5134901 27851913

[133] Increased number of regulatory T cells (T-regs) in the peripheral blood of patients with Her-2/neu-positive early breast cancer | SpringerLink (Accessed March 28, 2023). 10.1007/s00432-012-1258-3 PMC11824674 22760213

[134] Wang L Simons DL Lu X Tu TY Solomon S Wang R. Connecting blood and intratumoral Treg cell activity in predicting future relapse in breast cancer. Nat Immunol. (2019) 20:1220–30. doi: 10.1038/s41590-019-0429-7 PMC8802768 31285626

[135] Kos K Aslam MA van de Ven R Wellenstein MD Pieters W van Weverwijk A. Tumor-educated Tregs drive organ-specific metastasis in breast cancer by impairing NK cells in the lymph node niche. Cell Rep. (2022) 38:110447. doi: 10.1016/j.celrep.2022.110447 35235800

[136] Breast cancer tumor microenvironment affects Treg/IL-17-producing Treg/Th17 cell axis: Molecular and therapeutic perspectives - ScienceDirect. Available online at: https://www.sciencedirect.com/science/article/pii/S2372770523000037bib284 (Accessed March 28, 2023). 10.1016/j.omto.2023.01.001 PMC9922830 36816749

[137] Malla RR Vasudevaraju P Vempati RK Rakshmitha M Merchant N Nagaraju GP. Regulatory T cells: Their role in triple-negative breast cancer progression and metastasis. Cancer. (2022) 128:1171–83. doi: 10.1002/cncr.34084 34990009

[138] Oshi M Asaoka M Tokumaru Y Angarita FA Yan L Matsuyama R. Abundance of regulatory T cell (Treg) as a predictive biomarker for neoadjuvant chemotherapy in triple-negative breast cancer. Cancers (Basel). (2020) 12:3038. doi: 10.3390/cancers12103038 33086518 PMC7603157

[139] Akinleye A Rasool Z. Immune checkpoint inhibitors of PD-L1 as cancer therapeutics. J Hematol Oncol. (2019) 12:92. doi: 10.1186/s13045-019-0779-5 31488176 PMC6729004

[140] Nakasone E Hurvitz S McCann K. Harnessing the immune system in the battle against breast cancer. Drugs Context. (2018) 7:1–21. doi: 10.7573/dic.212520 PMC5810622 29456568

[141] Bewersdorf JP Shallis RM Zeidan AM. Immune checkpoint inhibition in myeloid Malignancies: Moving beyond the PD-1/PD-L1 and CTLA-4 pathways. Blood Rev. (2021) 45:100709. doi: 10.1016/j.blre.2020.100709 32487480

[142] Li Y Miao W He D Wang S Lou J Jiang Y. Recent progress on immunotherapy for breast cancer: tumor microenvironment, nanotechnology and more. Front Bioengineering Biotechnol. (2021) 9:680315. doi: 10.3389/fbioe.2021.680315 PMC8207056 34150736

[143] Yi M Jiao D Xu H Liu Q Zhao W Han X. Biomarkers for predicting efficacy of PD-1/PD-L1 inhibitors. Mol Cancer. (2018) 17:129. doi: 10.1186/s12943-018-0864-3 30139382 PMC6107958

[144] Dong X Dai H Lin Y Sheng X Li Y Wang Y. TIMELESS upregulates PD-L1 expression and exerts an immunosuppressive role in breast cancer. J Trans Med. (2023) 21:400. doi: 10.1186/s12967-023-04257-6 PMC10280842 37340461

[145] Dong P Xiong Y Yue J Hanley SJB Watari H. Tumor-intrinsic PD-L1 signaling in cancer initiation, development and treatment: beyond immune evasion. Front Oncol. (2018) 8:386. doi: 10.3389/fonc.2018.00386 30283733 PMC6156376

[146] Zhao R Song Y Wang Y Huang Y Li Z Cui Y. PD-1/PD-L1 blockade rescue exhausted CD8+ T cells in gastrointestinal stromal tumours via the PI3K/Akt/mTOR signalling pathway. Cell Prolif. (2019) 52:e12571. doi: 10.1111/cpr.12571 30714229 PMC6536456

[147] Ye F Dewanjee S Li Y Jha NK Chen Z-S Kumar A. Advancements in clinical aspects of targeted therapy and immunotherapy in breast cancer. Mol Cancer. (2023) 22:105. doi: 10.1186/s12943-023-01805-y 37415164 PMC10324146

[148] Shah M Osgood CL Amatya AK Fiero MH Pierce WF Nair A. FDA approval summary: pembrolizumab for neoadjuvant and adjuvant treatment of patients with high-risk early-stage triple-negative breast cancer. Clin Cancer Res. (2022) 28:5249–53. doi: 10.1158/1078-0432.CCR-22-1110 35925043

[149] Adams S Loi S Toppmeyer D Cescon DW De Laurentiis M Nanda R. Pembrolizumab monotherapy for previously untreated, PD-L1-positive, metastatic triple-negative breast cancer: cohort B of the phase II KEYNOTE-086 study. Ann Oncol. (2019) 30:405–411. doi: 10.1093/annonc/mdy518 30475947

[150] Valenza C Rizzo G Passalacqua MI Boldrini L Corti C Trapani D. Evolving treatment landscape of immunotherapy in breast cancer: current issues and future perspectives. Ther Adv Med Oncol. (2023) 15:17588359221146129. doi: 10.1177/17588359221146129 36743524 PMC9893403

[151] Kok M Voorwerk L Horlings H Sikorska K van der Vijver K Slagter M. Adaptive phase II randomized trial of nivolumab after induction treatment in triple negative breast cancer (TONIC trial): Final response data stage I and first translational data. JCO. (2018) 36:1012–2. doi: 10.1200/JCO.2018.36.15_suppl.1012

[152] Planes-Laine G Rochigneux P Bertucci F Chrétien A-S Viens P Sabatier R. PD-1/PD-L1 targeting in breast cancer: the first clinical evidences are emerging. A literature review. Cancers (Basel). (2019) 11:1033. doi: 10.3390/cancers11071033 31336685 PMC6679223

[153] Dirix LY Takacs I Jerusalem G Nikolinakos P Arkenau H-T Forero-Torres A. Avelumab, an anti-PD-L1 antibody, in patients with locally advanced or metastatic breast cancer: a phase 1b JAVELIN Solid Tumor study. Breast Cancer Res Treat. (2018) 167:671–86. doi: 10.1007/s10549-017-4537-5 PMC5807460 29063313

[154] Conte PF Dieci MV Bisagni G De Laurentiis M Tondini CA Schmid P. Phase III randomized study of adjuvant treatment with the ANTI-PD-L1 antibody avelumab for high-risk triple negative breast cancer patients: The A-BRAVE trial. JCO. (2020) 38:TPS598–8. doi: 10.1200/JCO.2020.38.15_suppl.TPS598

[155] Emens LA Cruz C Eder JP Braiteh F Chung C Tolaney SM. Long-term clinical outcomes and biomarker analyses of atezolizumab therapy for patients with metastatic triple-negative breast cancer: A phase 1 study. JAMA Oncol. (2019) 5:74–82. doi: 10.1001/jamaoncol.2018.4224 30242306 PMC6439773

[156] Adams S Diamond JR Hamilton E Pohlmann PR Tolaney SM Chang C-W. Atezolizumab plus nab-paclitaxel in the treatment of metastatic triple-negative breast cancer with 2-year survival follow-up. JAMA Oncol. (2019) 5:334–42. doi: 10.1001/jamaoncol.2018.5152 PMC6439843 30347025

[157] Gianni L Huang CS Egle D Bermejo B Zamagni C Thill M. Pathologic complete response (pCR) to neoadjuvant treatment with or without atezolizumab in triple-negative, early high-risk and locally advanced breast cancer: NeoTRIP Michelangelo randomized study. Ann Oncol. (2022) 33:534–43. doi: 10.1016/j.annonc.2022.02.004 35182721

[158] Domchek SM Postel-Vinay S Im S-A Park YH Delord J-P Italiano A. Olaparib and durvalumab in patients with germline BRCA-mutated metastatic breast cancer (MEDIOLA): an open-label, multicentre, phase 1/2, basket study. Lancet Oncol. (2020) 21:1155–64. doi: 10.1016/S1470-2045(20)30324-7 32771088

[159] Loibl S Untch M Burchardi N Huober J Sinn BV Blohmer J-U. A randomised phase II study investigating durvalumab in addition to an anthracycline taxane-based neoadjuvant therapy in early triple-negative breast cancer: clinical results and biomarker analysis of GeparNuevo study. Ann Oncol. (2019) 30:1279–88. doi: 10.1093/annonc/mdz158 31095287

[160] Bachelot T Filleron T Bieche I Arnedos M Campone M Dalenc F. Durvalumab compared to maintenance chemotherapy in metastatic breast cancer: the randomized phase II SAFIR02-BREAST IMMUNO trial. Nat Med. (2021) 27:250–5. doi: 10.1038/s41591-020-01189-2 33462450

[161] Comin-Anduix B Escuin-Ordinas H Ibarrondo FJ. Tremelimumab: research and clinical development. OTT. (2016) 9:1767–76. doi: 10.2147/OTT.S65802 PMC4809326 27042127

[162] Page DB Beal K Linch SN Spinelli KJ Rodine M Halpenny D. Brain radiotherapy, tremelimumab-mediated CTLA-4-directed blockade +/- trastuzumab in patients with breast cancer brain metastases. NPJ Breast Cancer. (2022) 8:50. doi: 10.1038/s41523-022-00404-2 35440655 PMC9018738

[163] Nguyen VP Campbell KM Nowicki TS Elumalai N Medina E Baselga-Carretero I. A pilot study of neoadjuvant nivolumab, ipilimumab, and intralesional oncolytic virotherapy for HER2-negative breast cancer. Cancer Res Commun. (2023) 3:1628–37. doi: 10.1158/2767-9764.CRC-23-0145 PMC10445661 37621406

[164] Zhou H Fu X Li Q Niu T. Safety and efficacy of anti-PD-1 monoclonal antibodies in patients with relapsed or refractory lymphoma: A meta-analysis of prospective clinic trails. Front Pharmacol. (2019) 10:387. doi: 10.3389/fphar.2019.00387 31118893 PMC6504777

[165] Adams S Loi S Toppmeyer D Cescon DW De Laurentiis M Nanda R. Pembrolizumab monotherapy for previously untreated, PD-L1-positive, metastatic triple-negative breast cancer: cohort B of the phase II KEYNOTE-086 study. Ann Oncol. (2019) 30:405–11. doi: 10.1093/annonc/mdy518 30475947

[166] Wu Z Man S Sun R Li Z Wu Y Zuo D. Recent advances and challenges of immune checkpoint inhibitors in immunotherapy of non-small cell lung cancer. Int Immunopharmacol. (2020) 85:106613. doi: 10.1016/j.intimp.2020.106613 32450531

[167] Keilholz U Mehnert JM Bauer S Bourgeois H Patel MR Gravenor D. Avelumab in patients with previously treated metastatic melanoma: phase 1b results from the JAVELIN Solid Tumor trial. J Immunother Cancer. (2019) 7:12. doi: 10.1186/s40425-018-0459-y 30651126 PMC6335739

[168] Blevins DJ Hanley R Bolduc T Powell DA Gignac M Walker K. *In vitro* assessment of putative PD-1/PD-L1 inhibitors: suggestions of an alternative mode of action. ACS Med Chem Lett. (2019) 10:1187–92. doi: 10.1021/acsmedchemlett.9b00221 PMC6691557 31413804

[169] Le Tourneau C Hoimes C Zarwan C Wong DJ Bauer S Claus R. Avelumab in patients with previously treated metastatic adrenocortical carcinoma: phase 1b results from the JAVELIN solid tumor trial. J Immunother Cancer. (2018) 6:111. doi: 10.1186/s40425-018-0424-9 30348224 PMC6198369

[170] Adams S Diamond JR Hamilton E Pohlmann PR Tolaney SM Chang C-W. Atezolizumab plus nab-paclitaxel in the treatment of metastatic triple-negative breast cancer with 2-year survival follow-up: A phase 1b clinical trial. JAMA Oncol. (2019) 5:334–42. doi: 10.1001/jamaoncol.2018.5152 PMC6439843 30347025

[171] Turner NC Ro J André F Loi S Verma S Iwata H. Palbociclib in hormone-receptor-positive advanced breast cancer. N Engl J Med. (2015) 373:209–19. doi: 10.1056/NEJMoa1505270 26030518

[172] Dean JL Thangavel C McClendon AK Reed CA Knudsen ES. Therapeutic CDK4/6 inhibition in breast cancer: key mechanisms of response and failure. Oncogene. (2010) 29:4018–32. doi: 10.1038/onc.2010.154 20473330

[173] Finn RS Dering J Conklin D Kalous O Cohen DJ Desai AJ. PD 0332991, a selective cyclin D kinase 4/6 inhibitor, preferentially inhibits proliferation of luminal estrogen receptor-positive human breast cancer cell lines in *vitro* . Breast Cancer Res. (2009) 11:R77. doi: 10.1186/bcr2419 19874578 PMC2790859

[174] Marty B Maire V Gravier E Rigaill G Vincent-Salomon A Kappler M. Frequent PTEN genomic alterations and activated phosphatidylinositol 3-kinase pathway in basal-like breast cancer cells. Breast Cancer Res. (2008) 10:R101. doi: 10.1186/bcr2204 19055754 PMC2656897

[175] Subbiah IM Lei X Weinberg JS Sulman EP Chavez-MacGregor M Tripathy D. Validation and development of a modified breast graded prognostic assessment as a tool for survival in patients with breast cancer and brain metastases. J Clin Oncol. (2015) 33:2239–45. doi: 10.1200/JCO.2014.58.8517 PMC5098846 25987700

[176] Sorlie T Tibshirani R Parker J Hastie T Marron JS Nobel A. Repeated observation of breast tumor subtypes in independent gene expression data sets. Proc Natl Acad Sci U.S.A. (2003) 100:8418–23. doi: 10.1073/pnas.0932692100 PMC166244 12829800

[177] Antonia S Goldberg SB Balmanoukian A Chaft JE Sanborn RE Gupta A. Safety and antitumour activity of durvalumab plus tremelimumab in non-small cell lung cancer: a multicentre, phase 1b study. Lancet Oncol. (2016) 17:299–308. doi: 10.1016/S1470-2045(15)00544-6 26858122 PMC5500167

[178] Huang X Song C Zhang J Zhu L Tang H. Circular RNAs in breast cancer diagnosis, treatment and prognosis. Oncol Res. (2023) 32:241–9. doi: 10.32604/or.2023.046582 PMC10765117 38186573

