# Erratum: Investigating tumor immunogenicity in breast cancer: deciphering the tumor immune response to enhance therapeutic approaches

**DOI:** 10.3389/fimmu.2024.1532921

**Published:** 2024-12-11

**Authors:** Frontiers Production Office

**Affiliations:** Frontiers Media SA, Lausanne, Switzerland

**Keywords:** breast cancer, immune microenviroment, immune response, therapy resistance, immunotherapy

Due to a production error, there was a mistake in **Figure 2** and **Figure 3** as published. The correct **Figure 2** was inadvertently published as **Figure 3**, and the correct **Figure 3** file was omitted from the published article. The corrected **Figure 2** and **Figure 3** appear below.

**Figure 2:**
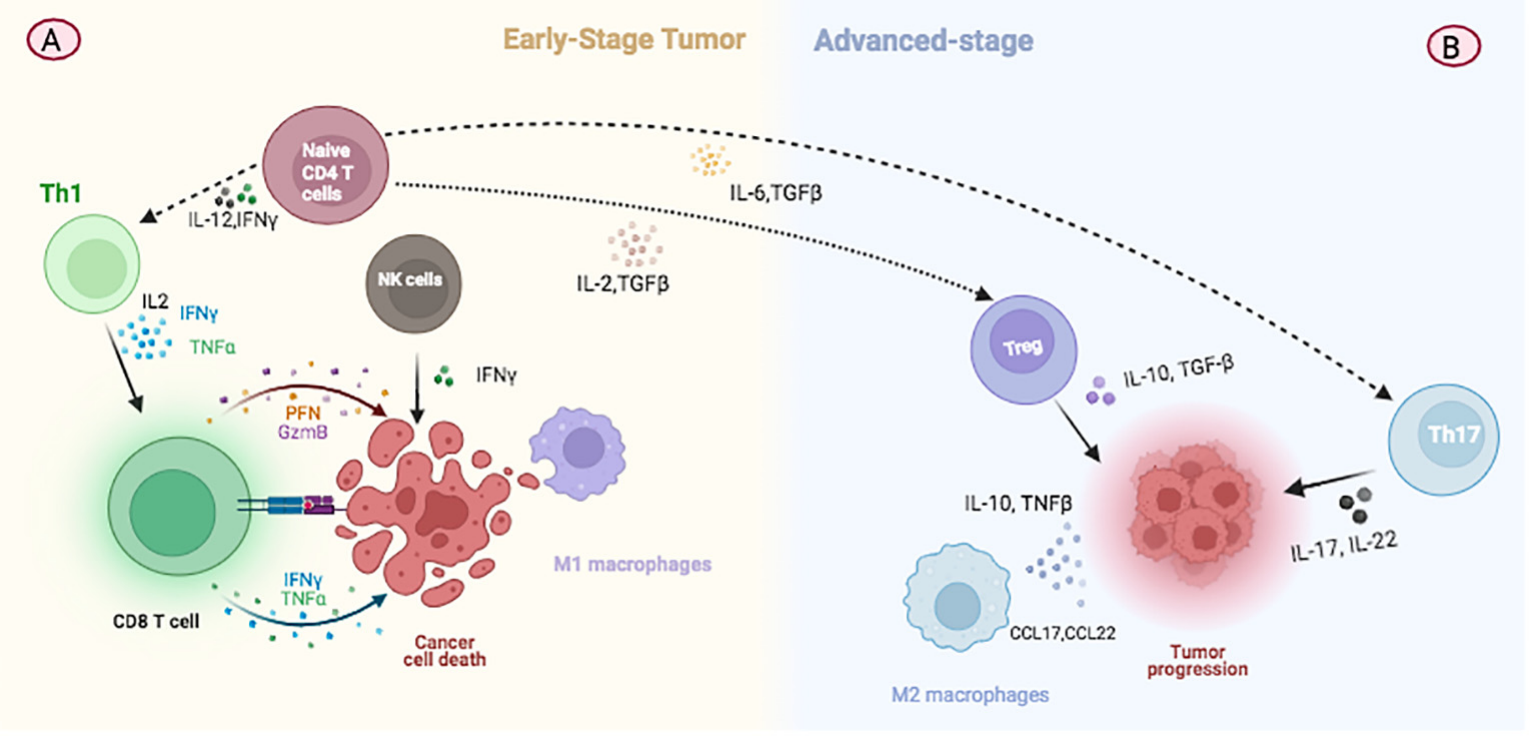
Illustration depicting changes in immune cell populations during breast cancer progression. **(A)** At the initial stage of tumor development, TILs predominantly consist of Th1 and CD8+ T cells which are involved in immunosurveillance and combating malignant cell growth. **(B)** In advanced stages of cancer, there is a notable increase in CD4+ TILs, with a shift towards the predominance of Treg and Th17 cells. These changes contribute to tumor growth by modulating the immune environment within the tumor.

**Figure 3:**
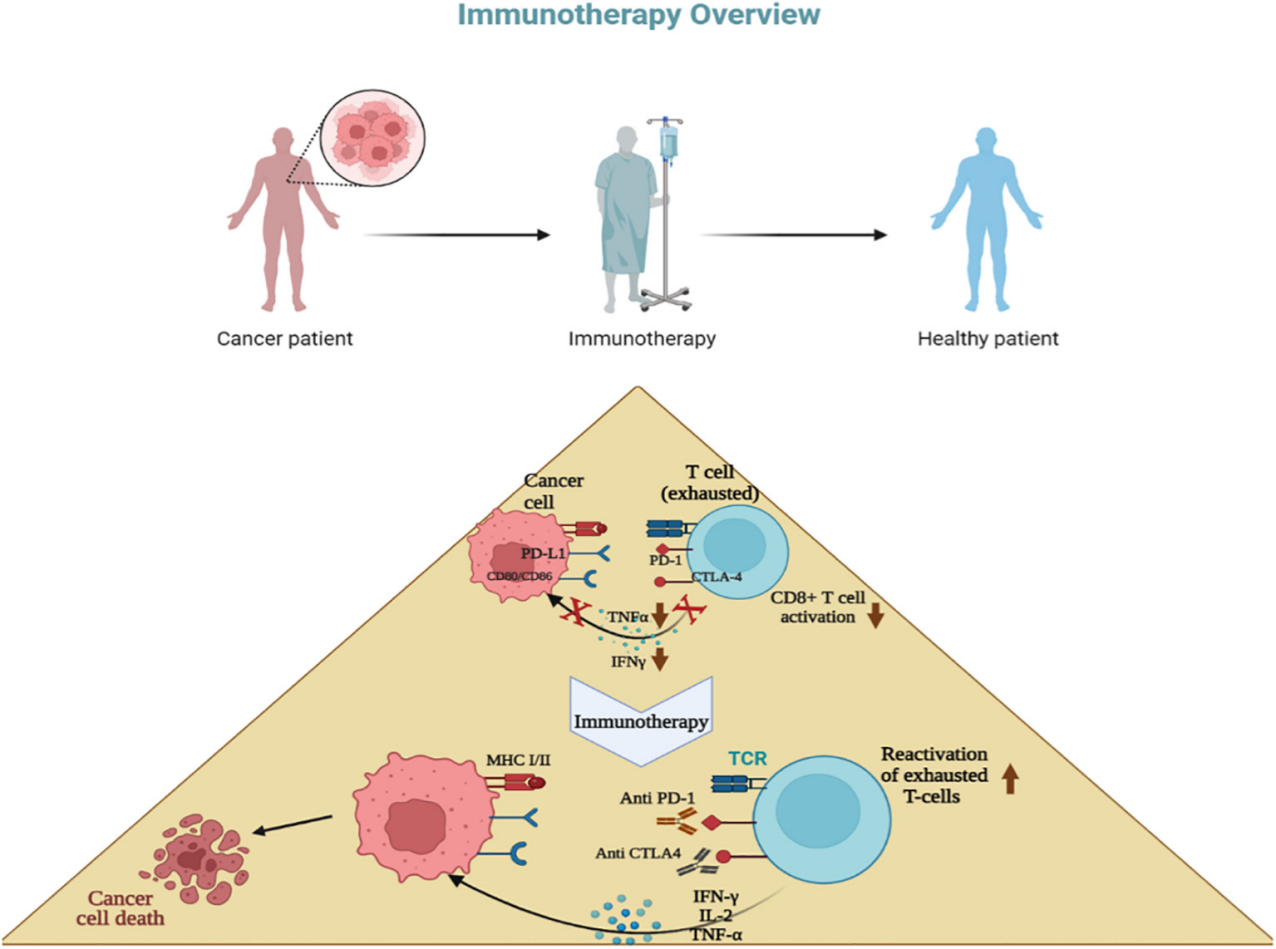
Concept of immunotherapy. T cells become exhausted after prolonged antigen stimulation and interaction with inhibitory ligands (PD-L1,L2; CD80,CD86) related to immune-checkpoint pathways. Immunotherapy involves inhibiting these immune checkpoint pathways using antibodies, with the goal of restoring T-cell functions. Most breast cancer immunotherapies focus mainly on anti-PD-1 drugs, which stop the interaction between PD-1, PD-L1, and PD-L2, such as pembrolizumab, avelumab, and atezolizumab.

The publisher apologizes for this mistake.

The original version of this article has been updated.

